# Supplementary material for: Prediction models of macro-nutrient content in plant organs of Cucumis melo in response to soil elements using support vector regression
Source: PeerJ. 2023 Oct 2;11:e15417. doi: 10.7717/peerj.15417 (PMC10552743; doi:10.7717/peerj.15417)
Supplement: Supplemental Information 1 — * Soil phosphorus and potassium content represent in mg kg−1. [file peerj-11-15417-s001.docx]

The chemical properties and elements composition in the soil, vermicompost, and manure samples used in the study represent according to the methodology described in Methods. The final data represents in Table S1.

**Table S1:**

**The chemical properties and elements composition in the soil, vermicompost, and manure samples used in the study.**

|  | South-eastern Iran  (Zahak county) | | | | |  | North-eastern Iran  (Fariman county) | | | | |
| --- | --- | --- | --- | --- | --- | --- | --- | --- | --- | --- | --- |
| Features | N | P | K | pH | EC |  | N | P | K | pH | EC |
|  | (%) | (%) | (%) | - | (dS m^-1^) |  | (%) | (%) | (%) | - | (dS m^-1^) |
| Soil | 0.03 | 16.6* | 170* | 8.12 | 3.2 |  | 0.058 | 39.5* | 193* | 7.62 | 5.02 |
| Cow manure | 1.14 | 0.71 | 1.10 | 8.02 | 3.50 |  | 1.33 | 0.65 | 1.01 | 7.50 | 3.26 |
| Sheep manure | 0.94 | 0.48 | 0.98 | 8.05 | 3.47 |  | 1.09 | 0.79 | 1.33 | 7.90 | 3.20 |
| Vermicompost | 1.40 | 1.02 | 1.10 | 8.25 | 7.5 |  | 1.50 | 1.30 | 1.20 | 7.30 | 6.40 |
| * Soil phosphorus and potassium content represent in mg kg^-1^. | | | | | | | | | | | |
