# Supplementary material for: Prediction models of macro-nutrient content in plant organs of Cucumis melo in response to soil elements using support vector regression
Source: PeerJ. 2023 Oct 2;11:e15417. doi: 10.7717/peerj.15417 (PMC10552743; doi:10.7717/peerj.15417)
Supplement: Supplemental Information 4 [file peerj-11-15417-s004.docx]

The statistical description of the predictive performance of fruit yield and nitrogen content in seeds, fruits, leaves, and roots for the training data (N = 144) represents according to the methodology described in Methods. The final data represents in Table S4.

**Table S4:**

**The statistical description of the predictive performance of fruit yield and nitrogen content in plant organs for the training data (N = 144).**

| Model N | RMSE | MAPE | RPD | R | R^2^ | Adjusted R^2^ | Standardized Beta | t | Sig. |
| --- | --- | --- | --- | --- | --- | --- | --- | --- | --- |
| Seed | 0.224 | 6.73% | 1.504 | 0.835^**^ | 0.697 | 0.695 | 0.835 | 18.060 | 0.000 |
| Fruit | 0.122 | 7.01% | 2.017 | 0.898^**^ | 0.807 | 0.805 | 0.898 | 24.345 | 0.000 |
| Leaf | 1.061 | 31.85% | 0.710 | 0.912^**^ | 0.832 | 0.831 | 0.912 | 26.519 | 0.000 |
| Root | 0.216 | 14.02% | 1.239 | 0.736^**^ | 0.542 | 0.539 | 0.736 | 12.970 | 0.000 |
| Fruit yield | 0.985 | 17.40% | 1.495 | 0.808^**^ | 0.652 | 0.650 | 0.808 | 16.318 | 0.000 |
