# Supplementary material for: Prediction models of macro-nutrient content in plant organs of Cucumis melo in response to soil elements using support vector regression
Source: PeerJ. 2023 Oct 2;11:e15417. doi: 10.7717/peerj.15417 (PMC10552743; doi:10.7717/peerj.15417)
Supplement: Supplemental Information 6 [file peerj-11-15417-s006.docx]

The statistical description of predicted values of fruit yield and phosphorus content in seeds, fruits, leaves, and roots presents according to the methodology described in Methods. The final data represents in Table S6.

**Table S6:**

**The statistical description of predicted values of fruit yield (kg per plant) and phosphorus content in plant organs (%).**

| Predicted P | N | Minimum | Maximum | Mean | S.D | C.V |
| --- | --- | --- | --- | --- | --- | --- |
| Seed | 144 | 15.18 | 44.55 | 23.915 | 5.822 | 0.2434 |
| Fruit | 144 | 10.34 | 40.83 | 22.955 | 6.375 | 0.2777 |
| Leaf | 144 | 14.55 | 32.85 | 23.966 | 4.782 | 0.1995 |
| Root | 144 | 9.77 | 55.49 | 21.705 | 7.816 | 0.3601 |
| Fruit yield | 144 | 1.87 | 5.68 | 3.471 | 1.259 | 0.3627 |
