# Supplementary material for: Prediction models of macro-nutrient content in plant organs of Cucumis melo in response to soil elements using support vector regression
Source: PeerJ. 2023 Oct 2;11:e15417. doi: 10.7717/peerj.15417 (PMC10552743; doi:10.7717/peerj.15417)
Supplement: Supplemental Information 7 [file peerj-11-15417-s007.docx]

The statistical description of the predictive performance of fruit yield and phosphorus content in seeds, fruits, leaves, and roots for the training data (N = 144) represents according to the methodology described in Methods. The final data represents in Table S7.

**Table S7:**

**The statistical description of the predictive performance of fruit yield and phosphorus content in plant organs for the training data (N = 144).**

| Model P | RMSE | MAPE | RPD | R | R^2^ | Adjusted R^2^ | Standardized Beta | t | Sig. |
| --- | --- | --- | --- | --- | --- | --- | --- | --- | --- |
| Seed | 0.334 | 0.46% | 17.455 | 0.998^**^ | 0.997 | 0.997 | 0.998 | 210.486 | 0.000 |
| Fruit | 0.228 | 0.38% | 27.957 | 0.999^**^ | 0.999 | 0.999 | 0.999 | 333.189 | 0.000 |
| Leaf | 22.98 | 90.57% | 0.208 | 0.991^**^ | 0.981 | 0.981 | 0.991 | 86.679 | 0.000 |
| Root | 0.579 | 0.82% | 13.493 | 0.997^**^ | 0.995 | 0.995 | 0.997 | 162.203 | 0.000 |
| Fruit yield | 0.485 | 9.55% | 2.597 | 0.942^**^ | 0.886 | 0.886 | 0.942 | 33.299 | 0.000 |
