# Supplementary material for: Prediction models of macro-nutrient content in plant organs of Cucumis melo in response to soil elements using support vector regression
Source: PeerJ. 2023 Oct 2;11:e15417. doi: 10.7717/peerj.15417 (PMC10552743; doi:10.7717/peerj.15417)
Supplement: Supplemental Information 10 [file peerj-11-15417-s010.docx]

The statistical description of the predictive performance of fruit yield and potassium content in seeds, fruits, leaves, and roots for the training data (N = 144) represents according to the methodology described in Methods. The final data represents in Table S10.

**Table S10:**

**The statistical description of the predictive performance of fruit yield and potassium content in plant organs for the training data (N = 144).**

| Model K | RMSE | MAPE | RPD | R | R^2^ | Adjusted R^2^ | Standardized Beta | t | Sig. |
| --- | --- | --- | --- | --- | --- | --- | --- | --- | --- |
| Seed | 0.681 | 2.39% | 3.577 | 0.970^**^ | 0.940 | 0.940 | 0.970 | 47.209 | 0.000 |
| Fruit | 0.465 | 1.77% | 5.174 | 0.984^**^ | 0.968 | 0.968 | 0.984 | 65.425 | 0.000 |
| Leaf | 12.148 | 132.11% | 0.148 | 0.992^**^ | 0.984 | 0.984 | 0.992 | 94.761 | 0.000 |
| Root | 0.703 | 1.97% | 4.420 | 0.976^**^ | 0.952 | 0.952 | 0.976 | 53.122 | 0.000 |
| Fruit yield | 0.858 | 15.86% | 1.637 | 0.848^**^ | 0.719 | 0.717 | 0.848 | 19.067 | 0.000 |
