# Supplementary material for: Prediction models of macro-nutrient content in plant organs of Cucumis melo in response to soil elements using support vector regression
Source: PeerJ. 2023 Oct 2;11:e15417. doi: 10.7717/peerj.15417 (PMC10552743; doi:10.7717/peerj.15417)
Supplement: Supplemental Information 11 [file peerj-11-15417-s011.docx]

The statistical description of the predictive performance of fruit yield and nitrogen content in seeds, fruits, leaves, and roots for the test data (N = 192) represents according to the methodology described in Methods. The final data represents in Table S11.

**Table S11:**

**The statistical description of the predictive performance of fruit yield and nitrogen content in plant organs for the test data (N = 192).**

| Model N | RMSE | MAPE | RPD | R | R^2^ | Adjusted R^2^ | Standardized Beta | t | Sig. |
| --- | --- | --- | --- | --- | --- | --- | --- | --- | --- |
| Seed | 0.193 | 5.05 | 7.41 | 0.991 | 0.982 | 0.982 | 0.991 | 101.85 | 0.000 |
| Fruit | 0.105 | 5.26 | 7.89 | 0.992 | 0.984 | 0.981 | 0.992 | 108.54 | 0.000 |
| Leaf | 1.34 | 31.77 | 1.25 | 0.986 | 0.972 | 0.972 | 0.986 | 81.66 | 0.000 |
| Root | 0.187 | 10.52 | 2.84 | 0.940 | 0.884 | 0.883 | 0.940 | 38.01 | 0.000 |
| Fruit yield | 0.853 | 13.05 | 1.54 | 0.813 | 0.661 | 0.659 | 0.813 | 19.23 | 0.000 |
