# Supplementary material for: Prediction models of macro-nutrient content in plant organs of Cucumis melo in response to soil elements using support vector regression
Source: PeerJ. 2023 Oct 2;11:e15417. doi: 10.7717/peerj.15417 (PMC10552743; doi:10.7717/peerj.15417)
Supplement: Supplemental Information 12 [file peerj-11-15417-s012.docx]

The statistical description of the predictive performance of fruit yield and phosphorus content in seeds, fruits, leaves, and roots for the test data (N = 192) represents according to the methodology described in Methods. The final data represents in Table S12.

**Table S12:**

**The statistical description of the predictive performance of fruit yield and phosphorus content in plant organs for the test data (N = 192).**

| Model P | RMSE | MAPE | RPD | R | R^2^ | Adjusted R^2^ | Standardized Beta | t | Sig. |
| --- | --- | --- | --- | --- | --- | --- | --- | --- | --- |
| Seed | 0.288 | 0.34 | 18.54 | 0.999 | 0.997 | 0.997 | 0.999 | 258.50 | 0.000 |
| Fruit | 0.197 | 0.29 | 29.94 | 0.999 | 0.999 | 0.999 | 0.999 | 412.88 | 0.000 |
| Leaf | 22.76 | 88.94 | 0.199 | 0.992 | 0.985 | 0.984 | 0.992 | 110.103 | 0.000 |
| Root | 0.501 | 0.61 | 13.95 | 0.997 | 0.995 | 0.995 | 0.997 | 194.04 | 0.000 |
| Fruit yield | 0.419 | 7.16 | 3.04 | 0.955 | 0.911 | 0.911 | 0.955 | 44.22 | 0.000 |
