# Supplementary material for: Prediction models of macro-nutrient content in plant organs of Cucumis melo in response to soil elements using support vector regression
Source: PeerJ. 2023 Oct 2;11:e15417. doi: 10.7717/peerj.15417 (PMC10552743; doi:10.7717/peerj.15417)
Supplement: Supplemental Information 13 [file peerj-11-15417-s013.docx]

The statistical description of the predictive performance of fruit yield and potassium content in seeds, fruits, leaves, and roots for the test data (N = 192) represents according to the methodology described in Methods. The final data represents in Table S13.

**Table S13:**

**The statistical description of the predictive performance of fruit yield and potassium content in plant organs for the test data (N = 192).**

| Model K | RMSE | MAPE | RPD | R | R^2^ | Adjusted R^2^ | Standardized Beta | t | Sig. |
| --- | --- | --- | --- | --- | --- | --- | --- | --- | --- |
| Seed | 0.589 | 1.79 | 4.13 | 0.976 | 0.952 | 0.951 | 0.976 | 61.22 | 0.000 |
| Fruit | 0.402 | 1.33 | 5.84 | 0.987 | 0.974 | 0.974 | 0.987 | 84.22 | 0.000 |
| Leaf | 12.39 | 145.11 | 0.153 | 0.995 | 0.989 | 0.989 | 0.995 | 133.40 | 0.000 |
| Root | 0.608 | 1.47 | 4.81 | 0.979 | 0.959 | 0.959 | 0.979 | 66.54 | 0.000 |
| Fruit yield | 0.743 | 11.89 | 1.73 | 0.859 | 0.737 | 0.736 | 0.859 | 23.08 | 0.000 |
