# Supplementary material for: Prediction models of macro-nutrient content in plant organs of Cucumis melo in response to soil elements using support vector regression
Source: PeerJ. 2023 Oct 2;11:e15417. doi: 10.7717/peerj.15417 (PMC10552743; doi:10.7717/peerj.15417)
Supplement: Supplemental Information 14 — To further evaluate the model’s generalizability, the developed SVR model was tested using 192 new datasets. [file peerj-11-15417-s014.docx]

DATASET ACTIVATE DataSet1.

DESCRIPTIVES VARIABLES=OSeedNitrogen PSeedNitrogen

/STATISTICS=MEAN SUM STDDEV MIN MAX.

**Descriptives**

| **Notes** | | |
| --- | --- | --- |
| Output Created | | 14-Mar-2023 02:32:54 |
| Comments | |  |
| Input | Data | C:\Users\ABAS\Documents\Abbas-Revision.sav |
|  | Active Dataset | DataSet1 |
|  | Filter | <none> |
|  | Weight | <none> |
|  | Split File | <none> |
|  | N of Rows in Working Data File | 192 |
| Missing Value Handling | Definition of Missing | User defined missing values are treated as missing. |
|  | Cases Used | All non-missing data are used. |
| Syntax | | DESCRIPTIVES VARIABLES=OSeedNitrogen PSeedNitrogen  /STATISTICS=MEAN SUM STDDEV MIN MAX. |
| Resources | Processor Time | 00 00:00:00.047 |
|  | Elapsed Time | 00 00:00:00.177 |

[DataSet1] C:\Users\ABAS\Documents\Abbas-Revision.sav

| **Descriptive Statistics** | | | | | | |
| --- | --- | --- | --- | --- | --- | --- |
|  | N | Minimum | Maximum | Sum | Mean | Std. Deviation |
| OSeedNitrogen | 192 | 1.44 | 7.43 | 531.94 | 2.7705 | 1.44809 |
| PSeedNitrogen | 192 | 1.39 | 7.43 | 529.84 | 2.7596 | 1.43921 |
| Valid N (listwise) | 192 |  |  |  |  |  |

DESCRIPTIVES VARIABLES=OFruitNitrogen PFruitNitrogen

/STATISTICS=MEAN SUM STDDEV MIN MAX.

**Descriptives**

| **Notes** | | |
| --- | --- | --- |
| Output Created | | 14-Mar-2023 02:35:36 |
| Comments | |  |
| Input | Data | C:\Users\ABAS\Documents\Abbas-Revision.sav |
|  | Active Dataset | DataSet1 |
|  | Filter | <none> |
|  | Weight | <none> |
|  | Split File | <none> |
|  | N of Rows in Working Data File | 192 |
| Missing Value Handling | Definition of Missing | User defined missing values are treated as missing. |
|  | Cases Used | All non-missing data are used. |
| Syntax | | DESCRIPTIVES VARIABLES=OFruitNitrogen PFruitNitrogen  /STATISTICS=MEAN SUM STDDEV MIN MAX. |
| Resources | Processor Time | 00 00:00:00.000 |
|  | Elapsed Time | 00 00:00:00.003 |

[DataSet1] C:\Users\ABAS\Documents\Abbas-Revision.sav

| **Descriptive Statistics** | | | | | | |
| --- | --- | --- | --- | --- | --- | --- |
|  | N | Minimum | Maximum | Sum | Mean | Std. Deviation |
| OFruitNitrogen | 192 | .79 | 4.34 | 306.64 | 1.5971 | .84187 |
| PFruitNitrogen | 192 | .68 | 4.34 | 305.87 | 1.5931 | .83640 |
| Valid N (listwise) | 192 |  |  |  |  |  |

DESCRIPTIVES VARIABLES=OLeafNitrogen PLeafNitrogen

/STATISTICS=MEAN SUM STDDEV MIN MAX.

**Descriptives**

| **Notes** | | |
| --- | --- | --- |
| Output Created | | 14-Mar-2023 02:36:05 |
| Comments | |  |
| Input | Data | C:\Users\ABAS\Documents\Abbas-Revision.sav |
|  | Active Dataset | DataSet1 |
|  | Filter | <none> |
|  | Weight | <none> |
|  | Split File | <none> |
|  | N of Rows in Working Data File | 192 |
| Missing Value Handling | Definition of Missing | User defined missing values are treated as missing. |
|  | Cases Used | All non-missing data are used. |
| Syntax | | DESCRIPTIVES VARIABLES=OLeafNitrogen PLeafNitrogen  /STATISTICS=MEAN SUM STDDEV MIN MAX. |
| Resources | Processor Time | 00 00:00:00.000 |
|  | Elapsed Time | 00 00:00:00.004 |

[DataSet1] C:\Users\ABAS\Documents\Abbas-Revision.sav

| **Descriptive Statistics** | | | | | | |
| --- | --- | --- | --- | --- | --- | --- |
|  | N | Minimum | Maximum | Sum | Mean | Std. Deviation |
| OLeafNitrogen | 192 | 2.17 | 9.19 | 742.92 | 3.8694 | 1.71067 |
| PLeafNitrogen | 192 | 2.13 | 9.19 | 747.67 | 3.8941 | 1.68478 |
| Valid N (listwise) | 192 |  |  |  |  |  |

DESCRIPTIVES VARIABLES=ORootNitrogen PRootNitrogen

/STATISTICS=MEAN SUM STDDEV MIN MAX.

**Descriptives**

| **Notes** | | |
| --- | --- | --- |
| Output Created | | 14-Mar-2023 02:36:23 |
| Comments | |  |
| Input | Data | C:\Users\ABAS\Documents\Abbas-Revision.sav |
|  | Active Dataset | DataSet1 |
|  | Filter | <none> |
|  | Weight | <none> |
|  | Split File | <none> |
|  | N of Rows in Working Data File | 192 |
| Missing Value Handling | Definition of Missing | User defined missing values are treated as missing. |
|  | Cases Used | All non-missing data are used. |
| Syntax | | DESCRIPTIVES VARIABLES=ORootNitrogen PRootNitrogen  /STATISTICS=MEAN SUM STDDEV MIN MAX. |
| Resources | Processor Time | 00 00:00:00.000 |
|  | Elapsed Time | 00 00:00:00.005 |

[DataSet1] C:\Users\ABAS\Documents\Abbas-Revision.sav

| **Descriptive Statistics** | | | | | | |
| --- | --- | --- | --- | --- | --- | --- |
|  | N | Minimum | Maximum | Sum | Mean | Std. Deviation |
| ORootNitrogen | 192 | .71 | 2.90 | 239.77 | 1.2488 | .54700 |
| PRootNitrogen | 192 | .66 | 2.90 | 237.22 | 1.2355 | .53333 |
| Valid N (listwise) | 192 |  |  |  |  |  |

DESCRIPTIVES VARIABLES=OFruitYieldNitrogen PFruitYieldNitrogen

/STATISTICS=MEAN SUM STDDEV MIN MAX.

**Descriptives**

| **Notes** | | |
| --- | --- | --- |
| Output Created | | 14-Mar-2023 02:40:49 |
| Comments | |  |
| Input | Data | C:\Users\ABAS\Documents\Abbas-Revision.sav |
|  | Active Dataset | DataSet1 |
|  | Filter | <none> |
|  | Weight | <none> |
|  | Split File | <none> |
|  | N of Rows in Working Data File | 192 |
| Missing Value Handling | Definition of Missing | User defined missing values are treated as missing. |
|  | Cases Used | All non-missing data are used. |
| Syntax | | DESCRIPTIVES VARIABLES=OFruitYieldNitrogen PFruitYieldNitrogen  /STATISTICS=MEAN SUM STDDEV MIN MAX. |
| Resources | Processor Time | 00 00:00:00.016 |
|  | Elapsed Time | 00 00:00:00.006 |

[DataSet1] C:\Users\ABAS\Documents\Abbas-Revision.sav

| **Descriptive Statistics** | | | | | | |
| --- | --- | --- | --- | --- | --- | --- |
|  | N | Minimum | Maximum | Sum | Mean | Std. Deviation |
| OFruitYieldNitrogen | 192 | 1.64 | 6.80 | 693.48 | 3.6119 | 1.29064 |
| PFruitYieldNitrogen | 192 | 2.08 | 6.66 | 752.53 | 3.9194 | 1.31684 |
| Valid N (listwise) | 192 |  |  |  |  |  |

DESCRIPTIVES VARIABLES=OSeedPhosphorus PSeedPhosphorus

/STATISTICS=MEAN SUM STDDEV MIN MAX.

**Descriptives**

| **Notes** | | |
| --- | --- | --- |
| Output Created | | 14-Mar-2023 02:41:36 |
| Comments | |  |
| Input | Data | C:\Users\ABAS\Documents\Abbas-Revision.sav |
|  | Active Dataset | DataSet1 |
|  | Filter | <none> |
|  | Weight | <none> |
|  | Split File | <none> |
|  | N of Rows in Working Data File | 192 |
| Missing Value Handling | Definition of Missing | User defined missing values are treated as missing. |
|  | Cases Used | All non-missing data are used. |
| Syntax | | DESCRIPTIVES VARIABLES=OSeedPhosphorus PSeedPhosphorus  /STATISTICS=MEAN SUM STDDEV MIN MAX. |
| Resources | Processor Time | 00 00:00:00.000 |
|  | Elapsed Time | 00 00:00:00.005 |

[DataSet1] C:\Users\ABAS\Documents\Abbas-Revision.sav

| **Descriptive Statistics** | | | | | | |
| --- | --- | --- | --- | --- | --- | --- |
|  | N | Minimum | Maximum | Sum | Mean | Std. Deviation |
| OSeedPhosphorus | 192 | 15.21 | 46.80 | 4640.69 | 24.1703 | 5.40135 |
| PSeedPhosphorus | 192 | 15.18 | 44.55 | 4640.65 | 24.1701 | 5.35851 |
| Valid N (listwise) | 192 |  |  |  |  |  |

DESCRIPTIVES VARIABLES=OFruitPhosphorus PFruitPhosphorus

/STATISTICS=MEAN SUM STDDEV MIN MAX.

**Descriptives**

| **Notes** | | |
| --- | --- | --- |
| Output Created | | 14-Mar-2023 02:42:47 |
| Comments | |  |
| Input | Data | C:\Users\ABAS\Documents\Abbas-Revision.sav |
|  | Active Dataset | DataSet1 |
|  | Filter | <none> |
|  | Weight | <none> |
|  | Split File | <none> |
|  | N of Rows in Working Data File | 192 |
| Missing Value Handling | Definition of Missing | User defined missing values are treated as missing. |
|  | Cases Used | All non-missing data are used. |
| Syntax | | DESCRIPTIVES VARIABLES=OFruitPhosphorus PFruitPhosphorus  /STATISTICS=MEAN SUM STDDEV MIN MAX. |
| Resources | Processor Time | 00 00:00:00.016 |
|  | Elapsed Time | 00 00:00:00.005 |

[DataSet1] C:\Users\ABAS\Documents\Abbas-Revision.sav

| **Descriptive Statistics** | | | | | | |
| --- | --- | --- | --- | --- | --- | --- |
|  | N | Minimum | Maximum | Sum | Mean | Std. Deviation |
| OFruitPhosphorus | 192 | 10.29 | 40.88 | 4466.47 | 23.2629 | 5.91893 |
| PFruitPhosphorus | 192 | 10.34 | 40.83 | 4464.61 | 23.2532 | 5.91365 |
| Valid N (listwise) | 192 |  |  |  |  |  |

DESCRIPTIVES VARIABLES=OLeafPhosphorus PLeafPhosphorus

/STATISTICS=MEAN SUM STDDEV MIN MAX.

**Descriptives**

| **Notes** | | |
| --- | --- | --- |
| Output Created | | 14-Mar-2023 02:43:04 |
| Comments | |  |
| Input | Data | C:\Users\ABAS\Documents\Abbas-Revision.sav |
|  | Active Dataset | DataSet1 |
|  | Filter | <none> |
|  | Weight | <none> |
|  | Split File | <none> |
|  | N of Rows in Working Data File | 192 |
| Missing Value Handling | Definition of Missing | User defined missing values are treated as missing. |
|  | Cases Used | All non-missing data are used. |
| Syntax | | DESCRIPTIVES VARIABLES=OLeafPhosphorus PLeafPhosphorus  /STATISTICS=MEAN SUM STDDEV MIN MAX. |
| Resources | Processor Time | 00 00:00:00.000 |
|  | Elapsed Time | 00 00:00:00.005 |

[DataSet1] C:\Users\ABAS\Documents\Abbas-Revision.sav

| **Descriptive Statistics** | | | | | | |
| --- | --- | --- | --- | --- | --- | --- |
|  | N | Minimum | Maximum | Sum | Mean | Std. Deviation |
| OLeafPhosphorus | 192 | 14.60 | 34.39 | 4700.25 | 24.4805 | 4.57304 |
| PLeafPhosphorus | 192 | 14.55 | 32.85 | 4694.88 | 24.4525 | 4.54776 |
| Valid N (listwise) | 192 |  |  |  |  |  |

DESCRIPTIVES VARIABLES=ORootPhosphorus PRootPhosphorus

/STATISTICS=MEAN SUM STDDEV MIN MAX.

**Descriptives**

| **Notes** | | |
| --- | --- | --- |
| Output Created | | 14-Mar-2023 02:43:26 |
| Comments | |  |
| Input | Data | C:\Users\ABAS\Documents\Abbas-Revision.sav |
|  | Active Dataset | DataSet1 |
|  | Filter | <none> |
|  | Weight | <none> |
|  | Split File | <none> |
|  | N of Rows in Working Data File | 192 |
| Missing Value Handling | Definition of Missing | User defined missing values are treated as missing. |
|  | Cases Used | All non-missing data are used. |
| Syntax | | DESCRIPTIVES VARIABLES=ORootPhosphorus PRootPhosphorus  /STATISTICS=MEAN SUM STDDEV MIN MAX. |
| Resources | Processor Time | 00 00:00:00.000 |
|  | Elapsed Time | 00 00:00:00.005 |

[DataSet1] C:\Users\ABAS\Documents\Abbas-Revision.sav

| **Descriptive Statistics** | | | | | | |
| --- | --- | --- | --- | --- | --- | --- |
|  | N | Minimum | Maximum | Sum | Mean | Std. Deviation |
| ORootPhosphorus | 192 | 9.72 | 56.55 | 4169.46 | 21.7159 | 7.06303 |
| PRootPhosphorus | 192 | 9.77 | 55.49 | 4172.57 | 21.7321 | 7.00140 |
| Valid N (listwise) | 192 |  |  |  |  |  |

DESCRIPTIVES VARIABLES=OFruitYieldPhosphorus PFruitYieldPhosphorus

/STATISTICS=MEAN SUM STDDEV MIN MAX.

**Descriptives**

| **Notes** | | |
| --- | --- | --- |
| Output Created | | 14-Mar-2023 02:43:52 |
| Comments | |  |
| Input | Data | C:\Users\ABAS\Documents\Abbas-Revision.sav |
|  | Active Dataset | DataSet1 |
|  | Filter | <none> |
|  | Weight | <none> |
|  | Split File | <none> |
|  | N of Rows in Working Data File | 192 |
| Missing Value Handling | Definition of Missing | User defined missing values are treated as missing. |
|  | Cases Used | All non-missing data are used. |
| Syntax | | DESCRIPTIVES VARIABLES=OFruitYieldPhosphorus PFruitYieldPhosphorus  /STATISTICS=MEAN SUM STDDEV MIN MAX. |
| Resources | Processor Time | 00 00:00:00.000 |
|  | Elapsed Time | 00 00:00:00.006 |

[DataSet1] C:\Users\ABAS\Documents\Abbas-Revision.sav

| **Descriptive Statistics** | | | | | | |
| --- | --- | --- | --- | --- | --- | --- |
|  | N | Minimum | Maximum | Sum | Mean | Std. Deviation |
| OFruitYieldPhosphorus | 192 | 1.64 | 6.80 | 698.10 | 3.6359 | 1.40056 |
| PFruitYieldPhosphorus | 192 | 1.87 | 5.95 | 694.57 | 3.6175 | 1.28001 |
| Valid N (listwise) | 192 |  |  |  |  |  |

DESCRIPTIVES VARIABLES=OSeedPotassium PSeedPotassium

/STATISTICS=MEAN SUM STDDEV MIN MAX.

**Descriptives**

| **Notes** | | |
| --- | --- | --- |
| Output Created | | 14-Mar-2023 02:44:31 |
| Comments | |  |
| Input | Data | C:\Users\ABAS\Documents\Abbas-Revision.sav |
|  | Active Dataset | DataSet1 |
|  | Filter | <none> |
|  | Weight | <none> |
|  | Split File | <none> |
|  | N of Rows in Working Data File | 192 |
| Missing Value Handling | Definition of Missing | User defined missing values are treated as missing. |
|  | Cases Used | All non-missing data are used. |
| Syntax | | DESCRIPTIVES VARIABLES=OSeedPotassium PSeedPotassium  /STATISTICS=MEAN SUM STDDEV MIN MAX. |
| Resources | Processor Time | 00 00:00:00.000 |
|  | Elapsed Time | 00 00:00:00.011 |

[DataSet1] C:\Users\ABAS\Documents\Abbas-Revision.sav

| **Descriptive Statistics** | | | | | | |
| --- | --- | --- | --- | --- | --- | --- |
|  | N | Minimum | Maximum | Sum | Mean | Std. Deviation |
| OSeedPotassium | 192 | 9.62 | 22.93 | 2800.32 | 14.5850 | 2.62091 |
| PSeedPotassium | 192 | 9.82 | 22.73 | 2790.18 | 14.5322 | 2.43456 |
| Valid N (listwise) | 192 |  |  |  |  |  |

DESCRIPTIVES VARIABLES=OFruitPotassium PFruitPotassium

/STATISTICS=MEAN SUM STDDEV MIN MAX.

**Descriptives**

| **Notes** | | |
| --- | --- | --- |
| Output Created | | 14-Mar-2023 02:44:53 |
| Comments | |  |
| Input | Data | C:\Users\ABAS\Documents\Abbas-Revision.sav |
|  | Active Dataset | DataSet1 |
|  | Filter | <none> |
|  | Weight | <none> |
|  | Split File | <none> |
|  | N of Rows in Working Data File | 192 |
| Missing Value Handling | Definition of Missing | User defined missing values are treated as missing. |
|  | Cases Used | All non-missing data are used. |
| Syntax | | DESCRIPTIVES VARIABLES=OFruitPotassium PFruitPotassium  /STATISTICS=MEAN SUM STDDEV MIN MAX. |
| Resources | Processor Time | 00 00:00:00.000 |
|  | Elapsed Time | 00 00:00:00.012 |

[DataSet1] C:\Users\ABAS\Documents\Abbas-Revision.sav

| **Descriptive Statistics** | | | | | | |
| --- | --- | --- | --- | --- | --- | --- |
|  | N | Minimum | Maximum | Sum | Mean | Std. Deviation |
| OFruitPotassium | 192 | 12.23 | 22.84 | 3258.08 | 16.9692 | 2.45520 |
| PFruitPotassium | 192 | 12.35 | 22.68 | 3251.30 | 16.9338 | 2.35461 |
| Valid N (listwise) | 192 |  |  |  |  |  |

DESCRIPTIVES VARIABLES=OLeafPotassium PLeafPotassium

/STATISTICS=MEAN SUM STDDEV MIN MAX.

**Descriptives**

| **Notes** | | |
| --- | --- | --- |
| Output Created | | 14-Mar-2023 02:45:10 |
| Comments | |  |
| Input | Data | C:\Users\ABAS\Documents\Abbas-Revision.sav |
|  | Active Dataset | DataSet1 |
|  | Filter | <none> |
|  | Weight | <none> |
|  | Split File | <none> |
|  | N of Rows in Working Data File | 192 |
| Missing Value Handling | Definition of Missing | User defined missing values are treated as missing. |
|  | Cases Used | All non-missing data are used. |
| Syntax | | DESCRIPTIVES VARIABLES=OLeafPotassium PLeafPotassium  /STATISTICS=MEAN SUM STDDEV MIN MAX. |
| Resources | Processor Time | 00 00:00:00.016 |
|  | Elapsed Time | 00 00:00:00.008 |

[DataSet1] C:\Users\ABAS\Documents\Abbas-Revision.sav

| **Descriptive Statistics** | | | | | | |
| --- | --- | --- | --- | --- | --- | --- |
|  | N | Minimum | Maximum | Sum | Mean | Std. Deviation |
| OLeafPotassium | 192 | 1.19 | 13.80 | 1737.99 | 9.0520 | 1.93000 |
| PLeafPotassium | 192 | 1.19 | 13.75 | 1737.57 | 9.0498 | 1.90115 |
| Valid N (listwise) | 192 |  |  |  |  |  |

DESCRIPTIVES VARIABLES=ORootPotassium PRootPotassium

/STATISTICS=MEAN SUM STDDEV MIN MAX.

**Descriptives**

| **Notes** | | |
| --- | --- | --- |
| Output Created | | 14-Mar-2023 02:45:28 |
| Comments | |  |
| Input | Data | C:\Users\ABAS\Documents\Abbas-Revision.sav |
|  | Active Dataset | DataSet1 |
|  | Filter | <none> |
|  | Weight | <none> |
|  | Split File | <none> |
|  | N of Rows in Working Data File | 192 |
| Missing Value Handling | Definition of Missing | User defined missing values are treated as missing. |
|  | Cases Used | All non-missing data are used. |
| Syntax | | DESCRIPTIVES VARIABLES=ORootPotassium PRootPotassium  /STATISTICS=MEAN SUM STDDEV MIN MAX. |
| Resources | Processor Time | 00 00:00:00.000 |
|  | Elapsed Time | 00 00:00:00.006 |

[DataSet1] C:\Users\ABAS\Documents\Abbas-Revision.sav

| **Descriptive Statistics** | | | | | | |
| --- | --- | --- | --- | --- | --- | --- |
|  | N | Minimum | Maximum | Sum | Mean | Std. Deviation |
| ORootPotassium | 192 | 5.42 | 19.02 | 2336.40 | 12.1688 | 2.98102 |
| PRootPotassium | 192 | 5.47 | 18.89 | 2320.53 | 12.0861 | 2.92739 |
| Valid N (listwise) | 192 |  |  |  |  |  |

DESCRIPTIVES VARIABLES=OFruitYieldPotassium PFruitYieldPotassium

/STATISTICS=MEAN SUM STDDEV MIN MAX.

**Descriptives**

| **Notes** | | |
| --- | --- | --- |
| Output Created | | 14-Mar-2023 02:45:46 |
| Comments | |  |
| Input | Data | C:\Users\ABAS\Documents\Abbas-Revision.sav |
|  | Active Dataset | DataSet1 |
|  | Filter | <none> |
|  | Weight | <none> |
|  | Split File | <none> |
|  | N of Rows in Working Data File | 192 |
| Missing Value Handling | Definition of Missing | User defined missing values are treated as missing. |
|  | Cases Used | All non-missing data are used. |
| Syntax | | DESCRIPTIVES VARIABLES=OFruitYieldPotassium PFruitYieldPotassium  /STATISTICS=MEAN SUM STDDEV MIN MAX. |
| Resources | Processor Time | 00 00:00:00.000 |
|  | Elapsed Time | 00 00:00:00.005 |

[DataSet1] C:\Users\ABAS\Documents\Abbas-Revision.sav

| **Descriptive Statistics** | | | | | | |
| --- | --- | --- | --- | --- | --- | --- |
|  | N | Minimum | Maximum | Sum | Mean | Std. Deviation |
| OFruitYieldPotassium | 192 | 1.64 | 6.80 | 695.04 | 3.6200 | 1.31744 |
| PFruitYieldPotassium | 192 | 1.85 | 6.25 | 747.58 | 3.8936 | 1.28679 |
| Valid N (listwise) | 192 |  |  |  |  |  |

REGRESSION

/MISSING LISTWISE

/STATISTICS COEFF OUTS R ANOVA

/CRITERIA=PIN(.05) POUT(.10)

/NOORIGIN

/DEPENDENT PSeedNitrogen

/METHOD=ENTER OSeedNitrogen.

**Regression**

| **Notes** | | |
| --- | --- | --- |
| Output Created | | 14-Mar-2023 02:51:34 |
| Comments | |  |
| Input | Data | C:\Users\ABAS\Documents\Abbas-Revision.sav |
|  | Active Dataset | DataSet1 |
|  | Filter | <none> |
|  | Weight | <none> |
|  | Split File | <none> |
|  | N of Rows in Working Data File | 192 |
| Missing Value Handling | Definition of Missing | User-defined missing values are treated as missing. |
|  | Cases Used | Statistics are based on cases with no missing values for any variable used. |
| Syntax | | REGRESSION  /MISSING LISTWISE  /STATISTICS COEFF OUTS R ANOVA  /CRITERIA=PIN(.05) POUT(.10)  /NOORIGIN  /DEPENDENT PSeedNitrogen  /METHOD=ENTER OSeedNitrogen. |
| Resources | Processor Time | 00 00:00:00.015 |
|  | Elapsed Time | 00 00:00:00.052 |
|  | Memory Required | 1996 bytes |
|  | Additional Memory Required for Residual Plots | 0 bytes |

[DataSet1] C:\Users\ABAS\Documents\Abbas-Revision.sav

| **Variables Entered/Removed^b^** | | | |
| --- | --- | --- | --- |
| Model | Variables Entered | Variables Removed | Method |
| 1 | OSeedNitrogen | . | Enter |
| a. All requested variables entered.  b. Dependent Variable: PSeedNitrogen | | | |

| **Model Summary** | | | | |
| --- | --- | --- | --- | --- |
| Model | R | R Square | Adjusted R Square | Std. Error of the Estimate |
| 1 | .991^a^ | .982 | .982 | .19352 |
| a. Predictors: (Constant), OSeedNitrogen | | | | |

| **ANOVA^b^** | | | | | | |
| --- | --- | --- | --- | --- | --- | --- |
| Model | | Sum of Squares | df | Mean Square | F | Sig. |
| 1 | Regression | 388.511 | 1 | 388.511 | 10374.461 | .000^a^ |
|  | Residual | 7.115 | 190 | .037 |  |  |
|  | Total | 395.626 | 191 |  |  |  |
| a. Predictors: (Constant), OSeedNitrogen  b. Dependent Variable: PSeedNitrogen | | | | | | |

| **Coefficients^a^** | | | | | | |
| --- | --- | --- | --- | --- | --- | --- |
| Model | | Unstandardized Coefficients | | Standardized Coefficients | t | Sig. |
|  |  | B | Std. Error | Beta |  |  |
| 1 | (Constant) | .031 | .030 |  | 1.024 | .307 |
|  | OSeedNitrogen | .985 | .010 | .991 | 101.855 | .000 |
| a. Dependent Variable: PSeedNitrogen | | | | | | |

REGRESSION

/MISSING LISTWISE

/STATISTICS COEFF OUTS R ANOVA

/CRITERIA=PIN(.05) POUT(.10)

/NOORIGIN

/DEPENDENT PFruitNitrogen

/METHOD=ENTER OFruitNitrogen.

**Regression**

| **Notes** | | |
| --- | --- | --- |
| Output Created | | 14-Mar-2023 02:56:17 |
| Comments | |  |
| Input | Data | C:\Users\ABAS\Documents\Abbas-Revision.sav |
|  | Active Dataset | DataSet1 |
|  | Filter | <none> |
|  | Weight | <none> |
|  | Split File | <none> |
|  | N of Rows in Working Data File | 192 |
| Missing Value Handling | Definition of Missing | User-defined missing values are treated as missing. |
|  | Cases Used | Statistics are based on cases with no missing values for any variable used. |
| Syntax | | REGRESSION  /MISSING LISTWISE  /STATISTICS COEFF OUTS R ANOVA  /CRITERIA=PIN(.05) POUT(.10)  /NOORIGIN  /DEPENDENT PFruitNitrogen  /METHOD=ENTER OFruitNitrogen. |
| Resources | Processor Time | 00 00:00:00.015 |
|  | Elapsed Time | 00 00:00:00.012 |
|  | Memory Required | 1996 bytes |
|  | Additional Memory Required for Residual Plots | 0 bytes |

[DataSet1] C:\Users\ABAS\Documents\Abbas-Revision.sav

| **Variables Entered/Removed^b^** | | | |
| --- | --- | --- | --- |
| Model | Variables Entered | Variables Removed | Method |
| 1 | OFruitNitrogen | . | Enter |
| a. All requested variables entered.  b. Dependent Variable: PFruitNitrogen | | | |

| **Model Summary** | | | | |
| --- | --- | --- | --- | --- |
| Model | R | R Square | Adjusted R Square | Std. Error of the Estimate |
| 1 | .992^a^ | .984 | .984 | .10565 |
| a. Predictors: (Constant), OFruitNitrogen | | | | |

| **ANOVA^b^** | | | | | | |
| --- | --- | --- | --- | --- | --- | --- |
| Model | | Sum of Squares | df | Mean Square | F | Sig. |
| 1 | Regression | 131.497 | 1 | 131.497 | 11781.332 | .000^a^ |
|  | Residual | 2.121 | 190 | .011 |  |  |
|  | Total | 133.618 | 191 |  |  |  |
| a. Predictors: (Constant), OFruitNitrogen  b. Dependent Variable: PFruitNitrogen | | | | | | |

| **Coefficients^a^** | | | | | | |
| --- | --- | --- | --- | --- | --- | --- |
| Model | | Unstandardized Coefficients | | Standardized Coefficients | t | Sig. |
|  |  | B | Std. Error | Beta |  |  |
| 1 | (Constant) | .019 | .016 |  | 1.161 | .247 |
|  | OFruitNitrogen | .986 | .009 | .992 | 108.542 | .000 |
| a. Dependent Variable: PFruitNitrogen | | | | | | |

REGRESSION

/MISSING LISTWISE

/STATISTICS COEFF OUTS R ANOVA

/CRITERIA=PIN(.05) POUT(.10)

/NOORIGIN

/DEPENDENT PLeafNitrogen

/METHOD=ENTER OLeafNitrogen.

**Regression**

| **Notes** | | |
| --- | --- | --- |
| Output Created | | 14-Mar-2023 02:56:38 |
| Comments | |  |
| Input | Data | C:\Users\ABAS\Documents\Abbas-Revision.sav |
|  | Active Dataset | DataSet1 |
|  | Filter | <none> |
|  | Weight | <none> |
|  | Split File | <none> |
|  | N of Rows in Working Data File | 192 |
| Missing Value Handling | Definition of Missing | User-defined missing values are treated as missing. |
|  | Cases Used | Statistics are based on cases with no missing values for any variable used. |
| Syntax | | REGRESSION  /MISSING LISTWISE  /STATISTICS COEFF OUTS R ANOVA  /CRITERIA=PIN(.05) POUT(.10)  /NOORIGIN  /DEPENDENT PLeafNitrogen  /METHOD=ENTER OLeafNitrogen. |
| Resources | Processor Time | 00 00:00:00.016 |
|  | Elapsed Time | 00 00:00:00.017 |
|  | Memory Required | 1996 bytes |
|  | Additional Memory Required for Residual Plots | 0 bytes |

[DataSet1] C:\Users\ABAS\Documents\Abbas-Revision.sav

| **Variables Entered/Removed^b^** | | | |
| --- | --- | --- | --- |
| Model | Variables Entered | Variables Removed | Method |
| 1 | OLeafNitrogen | . | Enter |
| a. All requested variables entered.  b. Dependent Variable: PLeafNitrogen | | | |

| **Model Summary** | | | | |
| --- | --- | --- | --- | --- |
| Model | R | R Square | Adjusted R Square | Std. Error of the Estimate |
| 1 | .986^a^ | .972 | .972 | .28114 |
| a. Predictors: (Constant), OLeafNitrogen | | | | |

| **ANOVA^b^** | | | | | | |
| --- | --- | --- | --- | --- | --- | --- |
| Model | | Sum of Squares | df | Mean Square | F | Sig. |
| 1 | Regression | 527.131 | 1 | 527.131 | 6669.359 | .000^a^ |
|  | Residual | 15.017 | 190 | .079 |  |  |
|  | Total | 542.148 | 191 |  |  |  |
| a. Predictors: (Constant), OLeafNitrogen  b. Dependent Variable: PLeafNitrogen | | | | | | |

| **Coefficients^a^** | | | | | | |
| --- | --- | --- | --- | --- | --- | --- |
| Model | | Unstandardized Coefficients | | Standardized Coefficients | t | Sig. |
|  |  | B | Std. Error | Beta |  |  |
| 1 | (Constant) | .136 | .050 |  | 2.713 | .007 |
|  | OLeafNitrogen | .971 | .012 | .986 | 81.666 | .000 |
| a. Dependent Variable: PLeafNitrogen | | | | | | |

REGRESSION

/MISSING LISTWISE

/STATISTICS COEFF OUTS R ANOVA

/CRITERIA=PIN(.05) POUT(.10)

/NOORIGIN

/DEPENDENT PRootNitrogen

/METHOD=ENTER ORootNitrogen.

**Regression**

| **Notes** | | |
| --- | --- | --- |
| Output Created | | 14-Mar-2023 02:56:57 |
| Comments | |  |
| Input | Data | C:\Users\ABAS\Documents\Abbas-Revision.sav |
|  | Active Dataset | DataSet1 |
|  | Filter | <none> |
|  | Weight | <none> |
|  | Split File | <none> |
|  | N of Rows in Working Data File | 192 |
| Missing Value Handling | Definition of Missing | User-defined missing values are treated as missing. |
|  | Cases Used | Statistics are based on cases with no missing values for any variable used. |
| Syntax | | REGRESSION  /MISSING LISTWISE  /STATISTICS COEFF OUTS R ANOVA  /CRITERIA=PIN(.05) POUT(.10)  /NOORIGIN  /DEPENDENT PRootNitrogen  /METHOD=ENTER ORootNitrogen. |
| Resources | Processor Time | 00 00:00:00.000 |
|  | Elapsed Time | 00 00:00:00.016 |
|  | Memory Required | 1996 bytes |
|  | Additional Memory Required for Residual Plots | 0 bytes |

[DataSet1] C:\Users\ABAS\Documents\Abbas-Revision.sav

| **Variables Entered/Removed^b^** | | | |
| --- | --- | --- | --- |
| Model | Variables Entered | Variables Removed | Method |
| 1 | ORootNitrogen | . | Enter |
| a. All requested variables entered.  b. Dependent Variable: PRootNitrogen | | | |

| **Model Summary** | | | | |
| --- | --- | --- | --- | --- |
| Model | R | R Square | Adjusted R Square | Std. Error of the Estimate |
| 1 | .940^a^ | .884 | .883 | .18226 |
| a. Predictors: (Constant), ORootNitrogen | | | | |

| **ANOVA^b^** | | | | | | |
| --- | --- | --- | --- | --- | --- | --- |
| Model | | Sum of Squares | df | Mean Square | F | Sig. |
| 1 | Regression | 48.016 | 1 | 48.016 | 1445.377 | .000^a^ |
|  | Residual | 6.312 | 190 | .033 |  |  |
|  | Total | 54.328 | 191 |  |  |  |
| a. Predictors: (Constant), ORootNitrogen  b. Dependent Variable: PRootNitrogen | | | | | | |

| **Coefficients^a^** | | | | | | |
| --- | --- | --- | --- | --- | --- | --- |
| Model | | Unstandardized Coefficients | | Standardized Coefficients | t | Sig. |
|  |  | B | Std. Error | Beta |  |  |
| 1 | (Constant) | .091 | .033 |  | 2.765 | .006 |
|  | ORootNitrogen | .917 | .024 | .940 | 38.018 | .000 |
| a. Dependent Variable: PRootNitrogen | | | | | | |

REGRESSION

/MISSING LISTWISE

/STATISTICS COEFF OUTS R ANOVA

/CRITERIA=PIN(.05) POUT(.10)

/NOORIGIN

/DEPENDENT PFruitYieldNitrogen

/METHOD=ENTER OFruitYieldNitrogen.

**Regression**

| **Notes** | | |
| --- | --- | --- |
| Output Created | | 14-Mar-2023 02:57:21 |
| Comments | |  |
| Input | Data | C:\Users\ABAS\Documents\Abbas-Revision.sav |
|  | Active Dataset | DataSet1 |
|  | Filter | <none> |
|  | Weight | <none> |
|  | Split File | <none> |
|  | N of Rows in Working Data File | 192 |
| Missing Value Handling | Definition of Missing | User-defined missing values are treated as missing. |
|  | Cases Used | Statistics are based on cases with no missing values for any variable used. |
| Syntax | | REGRESSION  /MISSING LISTWISE  /STATISTICS COEFF OUTS R ANOVA  /CRITERIA=PIN(.05) POUT(.10)  /NOORIGIN  /DEPENDENT PFruitYieldNitrogen  /METHOD=ENTER OFruitYieldNitrogen. |
| Resources | Processor Time | 00 00:00:00.015 |
|  | Elapsed Time | 00 00:00:00.014 |
|  | Memory Required | 1996 bytes |
|  | Additional Memory Required for Residual Plots | 0 bytes |

[DataSet1] C:\Users\ABAS\Documents\Abbas-Revision.sav

| **Variables Entered/Removed^b^** | | | |
| --- | --- | --- | --- |
| Model | Variables Entered | Variables Removed | Method |
| 1 | OFruitYieldNitrogen | . | Enter |
| a. All requested variables entered.  b. Dependent Variable: PFruitYieldNitrogen | | | |

| **Model Summary** | | | | |
| --- | --- | --- | --- | --- |
| Model | R | R Square | Adjusted R Square | Std. Error of the Estimate |
| 1 | .813^a^ | .661 | .659 | .76909 |
| a. Predictors: (Constant), OFruitYieldNitrogen | | | | |

| **ANOVA^b^** | | | | | | |
| --- | --- | --- | --- | --- | --- | --- |
| Model | | Sum of Squares | df | Mean Square | F | Sig. |
| 1 | Regression | 218.822 | 1 | 218.822 | 369.947 | .000^a^ |
|  | Residual | 112.384 | 190 | .591 |  |  |
|  | Total | 331.206 | 191 |  |  |  |
| a. Predictors: (Constant), OFruitYieldNitrogen  b. Dependent Variable: PFruitYieldNitrogen | | | | | | |

| **Coefficients^a^** | | | | | | |
| --- | --- | --- | --- | --- | --- | --- |
| Model | | Unstandardized Coefficients | | Standardized Coefficients | t | Sig. |
|  |  | B | Std. Error | Beta |  |  |
| 1 | (Constant) | .924 | .165 |  | 5.589 | .000 |
|  | OFruitYieldNitrogen | .829 | .043 | .813 | 19.234 | .000 |
| a. Dependent Variable: PFruitYieldNitrogen | | | | | | |

REGRESSION

/MISSING LISTWISE

/STATISTICS COEFF OUTS R ANOVA

/CRITERIA=PIN(.05) POUT(.10)

/NOORIGIN

/DEPENDENT PSeedPhosphorus

/METHOD=ENTER OSeedPhosphorus.

**Regression**

| **Notes** | | |
| --- | --- | --- |
| Output Created | | 14-Mar-2023 02:57:53 |
| Comments | |  |
| Input | Data | C:\Users\ABAS\Documents\Abbas-Revision.sav |
|  | Active Dataset | DataSet1 |
|  | Filter | <none> |
|  | Weight | <none> |
|  | Split File | <none> |
|  | N of Rows in Working Data File | 192 |
| Missing Value Handling | Definition of Missing | User-defined missing values are treated as missing. |
|  | Cases Used | Statistics are based on cases with no missing values for any variable used. |
| Syntax | | REGRESSION  /MISSING LISTWISE  /STATISTICS COEFF OUTS R ANOVA  /CRITERIA=PIN(.05) POUT(.10)  /NOORIGIN  /DEPENDENT PSeedPhosphorus  /METHOD=ENTER OSeedPhosphorus. |
| Resources | Processor Time | 00 00:00:00.015 |
|  | Elapsed Time | 00 00:00:00.015 |
|  | Memory Required | 1996 bytes |
|  | Additional Memory Required for Residual Plots | 0 bytes |

[DataSet1] C:\Users\ABAS\Documents\Abbas-Revision.sav

| **Variables Entered/Removed^b^** | | | |
| --- | --- | --- | --- |
| Model | Variables Entered | Variables Removed | Method |
| 1 | OSeedPhosphorus | . | Enter |
| a. All requested variables entered.  b. Dependent Variable: PSeedPhosphorus | | | |

| **Model Summary** | | | | |
| --- | --- | --- | --- | --- |
| Model | R | R Square | Adjusted R Square | Std. Error of the Estimate |
| 1 | .999^a^ | .997 | .997 | .28607 |
| a. Predictors: (Constant), OSeedPhosphorus | | | | |

| **ANOVA^b^** | | | | | | |
| --- | --- | --- | --- | --- | --- | --- |
| Model | | Sum of Squares | df | Mean Square | F | Sig. |
| 1 | Regression | 5468.749 | 1 | 5468.749 | 66824.383 | .000^a^ |
|  | Residual | 15.549 | 190 | .082 |  |  |
|  | Total | 5484.298 | 191 |  |  |  |
| a. Predictors: (Constant), OSeedPhosphorus  b. Dependent Variable: PSeedPhosphorus | | | | | | |

| **Coefficients^a^** | | | | | | |
| --- | --- | --- | --- | --- | --- | --- |
| Model | | Unstandardized Coefficients | | Standardized Coefficients | t | Sig. |
|  |  | B | Std. Error | Beta |  |  |
| 1 | (Constant) | .226 | .095 |  | 2.376 | .018 |
|  | OSeedPhosphorus | .991 | .004 | .999 | 258.504 | .000 |
| a. Dependent Variable: PSeedPhosphorus | | | | | | |

REGRESSION

/MISSING LISTWISE

/STATISTICS COEFF OUTS R ANOVA

/CRITERIA=PIN(.05) POUT(.10)

/NOORIGIN

/DEPENDENT PFruitPhosphorus

/METHOD=ENTER OFruitPhosphorus.

**Regression**

| **Notes** | | |
| --- | --- | --- |
| Output Created | | 14-Mar-2023 02:58:19 |
| Comments | |  |
| Input | Data | C:\Users\ABAS\Documents\Abbas-Revision.sav |
|  | Active Dataset | DataSet1 |
|  | Filter | <none> |
|  | Weight | <none> |
|  | Split File | <none> |
|  | N of Rows in Working Data File | 192 |
| Missing Value Handling | Definition of Missing | User-defined missing values are treated as missing. |
|  | Cases Used | Statistics are based on cases with no missing values for any variable used. |
| Syntax | | REGRESSION  /MISSING LISTWISE  /STATISTICS COEFF OUTS R ANOVA  /CRITERIA=PIN(.05) POUT(.10)  /NOORIGIN  /DEPENDENT PFruitPhosphorus  /METHOD=ENTER OFruitPhosphorus. |
| Resources | Processor Time | 00 00:00:00.032 |
|  | Elapsed Time | 00 00:00:00.017 |
|  | Memory Required | 1996 bytes |
|  | Additional Memory Required for Residual Plots | 0 bytes |

[DataSet1] C:\Users\ABAS\Documents\Abbas-Revision.sav

| **Variables Entered/Removed^b^** | | | |
| --- | --- | --- | --- |
| Model | Variables Entered | Variables Removed | Method |
| 1 | OFruitPhosphorus | . | Enter |
| a. All requested variables entered.  b. Dependent Variable: PFruitPhosphorus | | | |

| **Model Summary** | | | | |
| --- | --- | --- | --- | --- |
| Model | R | R Square | Adjusted R Square | Std. Error of the Estimate |
| 1 | .999^a^ | .999 | .999 | .19783 |
| a. Predictors: (Constant), OFruitPhosphorus | | | | |

| **ANOVA^b^** | | | | | | |
| --- | --- | --- | --- | --- | --- | --- |
| Model | | Sum of Squares | df | Mean Square | F | Sig. |
| 1 | Regression | 6672.063 | 1 | 6672.063 | 170473.278 | .000^a^ |
|  | Residual | 7.436 | 190 | .039 |  |  |
|  | Total | 6679.499 | 191 |  |  |  |
| a. Predictors: (Constant), OFruitPhosphorus  b. Dependent Variable: PFruitPhosphorus | | | | | | |

| **Coefficients^a^** | | | | | | |
| --- | --- | --- | --- | --- | --- | --- |
| Model | | Unstandardized Coefficients | | Standardized Coefficients | t | Sig. |
|  |  | B | Std. Error | Beta |  |  |
| 1 | (Constant) | .024 | .058 |  | .413 | .680 |
|  | OFruitPhosphorus | .999 | .002 | .999 | 412.884 | .000 |
| a. Dependent Variable: PFruitPhosphorus | | | | | | |

REGRESSION

/MISSING LISTWISE

/STATISTICS COEFF OUTS R ANOVA

/CRITERIA=PIN(.05) POUT(.10)

/NOORIGIN

/DEPENDENT PLeafPhosphorus

/METHOD=ENTER OLeafPhosphorus.

**Regression**

| **Notes** | | |
| --- | --- | --- |
| Output Created | | 14-Mar-2023 02:58:45 |
| Comments | |  |
| Input | Data | C:\Users\ABAS\Documents\Abbas-Revision.sav |
|  | Active Dataset | DataSet1 |
|  | Filter | <none> |
|  | Weight | <none> |
|  | Split File | <none> |
|  | N of Rows in Working Data File | 192 |
| Missing Value Handling | Definition of Missing | User-defined missing values are treated as missing. |
|  | Cases Used | Statistics are based on cases with no missing values for any variable used. |
| Syntax | | REGRESSION  /MISSING LISTWISE  /STATISTICS COEFF OUTS R ANOVA  /CRITERIA=PIN(.05) POUT(.10)  /NOORIGIN  /DEPENDENT PLeafPhosphorus  /METHOD=ENTER OLeafPhosphorus. |
| Resources | Processor Time | 00 00:00:00.032 |
|  | Elapsed Time | 00 00:00:00.016 |
|  | Memory Required | 1996 bytes |
|  | Additional Memory Required for Residual Plots | 0 bytes |

[DataSet1] C:\Users\ABAS\Documents\Abbas-Revision.sav

| **Variables Entered/Removed^b^** | | | |
| --- | --- | --- | --- |
| Model | Variables Entered | Variables Removed | Method |
| 1 | OLeafPhosphorus | . | Enter |
| a. All requested variables entered.  b. Dependent Variable: PLeafPhosphorus | | | |

| **Model Summary** | | | | |
| --- | --- | --- | --- | --- |
| Model | R | R Square | Adjusted R Square | Std. Error of the Estimate |
| 1 | .992^a^ | .985 | .984 | .56642 |
| a. Predictors: (Constant), OLeafPhosphorus | | | | |

| **ANOVA^b^** | | | | | | |
| --- | --- | --- | --- | --- | --- | --- |
| Model | | Sum of Squares | df | Mean Square | F | Sig. |
| 1 | Regression | 3889.333 | 1 | 3889.333 | 12122.694 | .000^a^ |
|  | Residual | 60.958 | 190 | .321 |  |  |
|  | Total | 3950.291 | 191 |  |  |  |
| a. Predictors: (Constant), OLeafPhosphorus  b. Dependent Variable: PLeafPhosphorus | | | | | | |

| **Coefficients^a^** | | | | | | |
| --- | --- | --- | --- | --- | --- | --- |
| Model | | Unstandardized Coefficients | | Standardized Coefficients | t | Sig. |
|  |  | B | Std. Error | Beta |  |  |
| 1 | (Constant) | .296 | .223 |  | 1.326 | .186 |
|  | OLeafPhosphorus | .987 | .009 | .992 | 110.103 | .000 |
| a. Dependent Variable: PLeafPhosphorus | | | | | | |

REGRESSION

/MISSING LISTWISE

/STATISTICS COEFF OUTS R ANOVA

/CRITERIA=PIN(.05) POUT(.10)

/NOORIGIN

/DEPENDENT PRootPhosphorus

/METHOD=ENTER ORootPhosphorus.

**Regression**

| **Notes** | | |
| --- | --- | --- |
| Output Created | | 14-Mar-2023 02:59:06 |
| Comments | |  |
| Input | Data | C:\Users\ABAS\Documents\Abbas-Revision.sav |
|  | Active Dataset | DataSet1 |
|  | Filter | <none> |
|  | Weight | <none> |
|  | Split File | <none> |
|  | N of Rows in Working Data File | 192 |
| Missing Value Handling | Definition of Missing | User-defined missing values are treated as missing. |
|  | Cases Used | Statistics are based on cases with no missing values for any variable used. |
| Syntax | | REGRESSION  /MISSING LISTWISE  /STATISTICS COEFF OUTS R ANOVA  /CRITERIA=PIN(.05) POUT(.10)  /NOORIGIN  /DEPENDENT PRootPhosphorus  /METHOD=ENTER ORootPhosphorus. |
| Resources | Processor Time | 00 00:00:00.016 |
|  | Elapsed Time | 00 00:00:00.017 |
|  | Memory Required | 1996 bytes |
|  | Additional Memory Required for Residual Plots | 0 bytes |

[DataSet1] C:\Users\ABAS\Documents\Abbas-Revision.sav

| **Variables Entered/Removed^b^** | | | |
| --- | --- | --- | --- |
| Model | Variables Entered | Variables Removed | Method |
| 1 | ORootPhosphorus | . | Enter |
| a. All requested variables entered.  b. Dependent Variable: PRootPhosphorus | | | |

| **Model Summary** | | | | |
| --- | --- | --- | --- | --- |
| Model | R | R Square | Adjusted R Square | Std. Error of the Estimate |
| 1 | .997^a^ | .995 | .995 | .49739 |
| a. Predictors: (Constant), ORootPhosphorus | | | | |

| **ANOVA^b^** | | | | | | |
| --- | --- | --- | --- | --- | --- | --- |
| Model | | Sum of Squares | df | Mean Square | F | Sig. |
| 1 | Regression | 9315.750 | 1 | 9315.750 | 37654.544 | .000^a^ |
|  | Residual | 47.006 | 190 | .247 |  |  |
|  | Total | 9362.756 | 191 |  |  |  |
| a. Predictors: (Constant), ORootPhosphorus  b. Dependent Variable: PRootPhosphorus | | | | | | |

| **Coefficients^a^** | | | | | | |
| --- | --- | --- | --- | --- | --- | --- |
| Model | | Unstandardized Coefficients | | Standardized Coefficients | t | Sig. |
|  |  | B | Std. Error | Beta |  |  |
| 1 | (Constant) | .260 | .116 |  | 2.233 | .027 |
|  | ORootPhosphorus | .989 | .005 | .997 | 194.048 | .000 |
| a. Dependent Variable: PRootPhosphorus | | | | | | |

REGRESSION

/MISSING LISTWISE

/STATISTICS COEFF OUTS R ANOVA

/CRITERIA=PIN(.05) POUT(.10)

/NOORIGIN

/DEPENDENT PFruitYieldPhosphorus

/METHOD=ENTER OFruitYieldPhosphorus.

**Regression**

| **Notes** | | |
| --- | --- | --- |
| Output Created | | 14-Mar-2023 02:59:28 |
| Comments | |  |
| Input | Data | C:\Users\ABAS\Documents\Abbas-Revision.sav |
|  | Active Dataset | DataSet1 |
|  | Filter | <none> |
|  | Weight | <none> |
|  | Split File | <none> |
|  | N of Rows in Working Data File | 192 |
| Missing Value Handling | Definition of Missing | User-defined missing values are treated as missing. |
|  | Cases Used | Statistics are based on cases with no missing values for any variable used. |
| Syntax | | REGRESSION  /MISSING LISTWISE  /STATISTICS COEFF OUTS R ANOVA  /CRITERIA=PIN(.05) POUT(.10)  /NOORIGIN  /DEPENDENT PFruitYieldPhosphorus  /METHOD=ENTER OFruitYieldPhosphorus. |
| Resources | Processor Time | 00 00:00:00.016 |
|  | Elapsed Time | 00 00:00:00.014 |
|  | Memory Required | 1996 bytes |
|  | Additional Memory Required for Residual Plots | 0 bytes |

[DataSet1] C:\Users\ABAS\Documents\Abbas-Revision.sav

| **Variables Entered/Removed^b^** | | | |
| --- | --- | --- | --- |
| Model | Variables Entered | Variables Removed | Method |
| 1 | OFruitYieldPhosphorus | . | Enter |
| a. All requested variables entered.  b. Dependent Variable: PFruitYieldPhosphorus | | | |

| **Model Summary** | | | | |
| --- | --- | --- | --- | --- |
| Model | R | R Square | Adjusted R Square | Std. Error of the Estimate |
| 1 | .955^a^ | .911 | .911 | .38187 |
| a. Predictors: (Constant), OFruitYieldPhosphorus | | | | |

| **ANOVA^b^** | | | | | | |
| --- | --- | --- | --- | --- | --- | --- |
| Model | | Sum of Squares | df | Mean Square | F | Sig. |
| 1 | Regression | 285.231 | 1 | 285.231 | 1955.977 | .000^a^ |
|  | Residual | 27.707 | 190 | .146 |  |  |
|  | Total | 312.938 | 191 |  |  |  |
| a. Predictors: (Constant), OFruitYieldPhosphorus  b. Dependent Variable: PFruitYieldPhosphorus | | | | | | |

| **Coefficients^a^** | | | | | | |
| --- | --- | --- | --- | --- | --- | --- |
| Model | | Unstandardized Coefficients | | Standardized Coefficients | t | Sig. |
|  |  | B | Std. Error | Beta |  |  |
| 1 | (Constant) | .445 | .077 |  | 5.792 | .000 |
|  | OFruitYieldPhosphorus | .873 | .020 | .955 | 44.226 | .000 |
| a. Dependent Variable: PFruitYieldPhosphorus | | | | | | |

REGRESSION

/MISSING LISTWISE

/STATISTICS COEFF OUTS R ANOVA

/CRITERIA=PIN(.05) POUT(.10)

/NOORIGIN

/DEPENDENT PSeedPotassium

/METHOD=ENTER OSeedPotassium.

**Regression**

| **Notes** | | |
| --- | --- | --- |
| Output Created | | 14-Mar-2023 03:05:14 |
| Comments | |  |
| Input | Data | C:\Users\ABAS\Documents\Abbas-Revision.sav |
|  | Active Dataset | DataSet1 |
|  | Filter | <none> |
|  | Weight | <none> |
|  | Split File | <none> |
|  | N of Rows in Working Data File | 192 |
| Missing Value Handling | Definition of Missing | User-defined missing values are treated as missing. |
|  | Cases Used | Statistics are based on cases with no missing values for any variable used. |
| Syntax | | REGRESSION  /MISSING LISTWISE  /STATISTICS COEFF OUTS R ANOVA  /CRITERIA=PIN(.05) POUT(.10)  /NOORIGIN  /DEPENDENT PSeedPotassium  /METHOD=ENTER OSeedPotassium. |
| Resources | Processor Time | 00 00:00:00.015 |
|  | Elapsed Time | 00 00:00:00.014 |
|  | Memory Required | 1996 bytes |
|  | Additional Memory Required for Residual Plots | 0 bytes |

[DataSet1] C:\Users\ABAS\Documents\Abbas-Revision.sav

| **Variables Entered/Removed^b^** | | | |
| --- | --- | --- | --- |
| Model | Variables Entered | Variables Removed | Method |
| 1 | OSeedPotassium | . | Enter |
| a. All requested variables entered.  b. Dependent Variable: PSeedPotassium | | | |

| **Model Summary** | | | | |
| --- | --- | --- | --- | --- |
| Model | R | R Square | Adjusted R Square | Std. Error of the Estimate |
| 1 | .976^a^ | .952 | .951 | .53616 |
| a. Predictors: (Constant), OSeedPotassium | | | | |

| **ANOVA^b^** | | | | | | |
| --- | --- | --- | --- | --- | --- | --- |
| Model | | Sum of Squares | df | Mean Square | F | Sig. |
| 1 | Regression | 1077.454 | 1 | 1077.454 | 3748.121 | .000^a^ |
|  | Residual | 54.618 | 190 | .287 |  |  |
|  | Total | 1132.073 | 191 |  |  |  |
| a. Predictors: (Constant), OSeedPotassium  b. Dependent Variable: PSeedPotassium | | | | | | |

| **Coefficients^a^** | | | | | | |
| --- | --- | --- | --- | --- | --- | --- |
| Model | | Unstandardized Coefficients | | Standardized Coefficients | t | Sig. |
|  |  | B | Std. Error | Beta |  |  |
| 1 | (Constant) | 1.315 | .219 |  | 5.996 | .000 |
|  | OSeedPotassium | .906 | .015 | .976 | 61.222 | .000 |
| a. Dependent Variable: PSeedPotassium | | | | | | |

REGRESSION

/MISSING LISTWISE

/STATISTICS COEFF OUTS R ANOVA

/CRITERIA=PIN(.05) POUT(.10)

/NOORIGIN

/DEPENDENT PFruitPotassium

/METHOD=ENTER OFruitPotassium.

**Regression**

| **Notes** | | |
| --- | --- | --- |
| Output Created | | 14-Mar-2023 03:06:00 |
| Comments | |  |
| Input | Data | C:\Users\ABAS\Documents\Abbas-Revision.sav |
|  | Active Dataset | DataSet1 |
|  | Filter | <none> |
|  | Weight | <none> |
|  | Split File | <none> |
|  | N of Rows in Working Data File | 192 |
| Missing Value Handling | Definition of Missing | User-defined missing values are treated as missing. |
|  | Cases Used | Statistics are based on cases with no missing values for any variable used. |
| Syntax | | REGRESSION  /MISSING LISTWISE  /STATISTICS COEFF OUTS R ANOVA  /CRITERIA=PIN(.05) POUT(.10)  /NOORIGIN  /DEPENDENT PFruitPotassium  /METHOD=ENTER OFruitPotassium. |
| Resources | Processor Time | 00 00:00:00.016 |
|  | Elapsed Time | 00 00:00:00.017 |
|  | Memory Required | 1996 bytes |
|  | Additional Memory Required for Residual Plots | 0 bytes |

[DataSet1] C:\Users\ABAS\Documents\Abbas-Revision.sav

| **Variables Entered/Removed^b^** | | | |
| --- | --- | --- | --- |
| Model | Variables Entered | Variables Removed | Method |
| 1 | OFruitPotassium | . | Enter |
| a. All requested variables entered.  b. Dependent Variable: PFruitPotassium | | | |

| **Model Summary** | | | | |
| --- | --- | --- | --- | --- |
| Model | R | R Square | Adjusted R Square | Std. Error of the Estimate |
| 1 | .987^a^ | .974 | .974 | .38127 |
| a. Predictors: (Constant), OFruitPotassium | | | | |

| **ANOVA^b^** | | | | | | |
| --- | --- | --- | --- | --- | --- | --- |
| Model | | Sum of Squares | df | Mean Square | F | Sig. |
| 1 | Regression | 1031.321 | 1 | 1031.321 | 7094.519 | .000^a^ |
|  | Residual | 27.620 | 190 | .145 |  |  |
|  | Total | 1058.941 | 191 |  |  |  |
| a. Predictors: (Constant), OFruitPotassium  b. Dependent Variable: PFruitPotassium | | | | | | |

| **Coefficients^a^** | | | | | | |
| --- | --- | --- | --- | --- | --- | --- |
| Model | | Unstandardized Coefficients | | Standardized Coefficients | t | Sig. |
|  |  | B | Std. Error | Beta |  |  |
| 1 | (Constant) | .874 | .193 |  | 4.534 | .000 |
|  | OFruitPotassium | .946 | .011 | .987 | 84.229 | .000 |
| a. Dependent Variable: PFruitPotassium | | | | | | |

REGRESSION

/MISSING LISTWISE

/STATISTICS COEFF OUTS R ANOVA

/CRITERIA=PIN(.05) POUT(.10)

/NOORIGIN

/DEPENDENT PLeafPotassium

/METHOD=ENTER OLeafPotassium.

**Regression**

| **Notes** | | |
| --- | --- | --- |
| Output Created | | 14-Mar-2023 03:06:16 |
| Comments | |  |
| Input | Data | C:\Users\ABAS\Documents\Abbas-Revision.sav |
|  | Active Dataset | DataSet1 |
|  | Filter | <none> |
|  | Weight | <none> |
|  | Split File | <none> |
|  | N of Rows in Working Data File | 192 |
| Missing Value Handling | Definition of Missing | User-defined missing values are treated as missing. |
|  | Cases Used | Statistics are based on cases with no missing values for any variable used. |
| Syntax | | REGRESSION  /MISSING LISTWISE  /STATISTICS COEFF OUTS R ANOVA  /CRITERIA=PIN(.05) POUT(.10)  /NOORIGIN  /DEPENDENT PLeafPotassium  /METHOD=ENTER OLeafPotassium. |
| Resources | Processor Time | 00 00:00:00.016 |
|  | Elapsed Time | 00 00:00:00.021 |
|  | Memory Required | 1996 bytes |
|  | Additional Memory Required for Residual Plots | 0 bytes |

[DataSet1] C:\Users\ABAS\Documents\Abbas-Revision.sav

| **Variables Entered/Removed^b^** | | | |
| --- | --- | --- | --- |
| Model | Variables Entered | Variables Removed | Method |
| 1 | OLeafPotassium | . | Enter |
| a. All requested variables entered.  b. Dependent Variable: PLeafPotassium | | | |

| **Model Summary** | | | | |
| --- | --- | --- | --- | --- |
| Model | R | R Square | Adjusted R Square | Std. Error of the Estimate |
| 1 | .995^a^ | .989 | .989 | .19591 |
| a. Predictors: (Constant), OLeafPotassium | | | | |

| **ANOVA^b^** | | | | | | |
| --- | --- | --- | --- | --- | --- | --- |
| Model | | Sum of Squares | df | Mean Square | F | Sig. |
| 1 | Regression | 683.054 | 1 | 683.054 | 17796.272 | .000^a^ |
|  | Residual | 7.293 | 190 | .038 |  |  |
|  | Total | 690.347 | 191 |  |  |  |
| a. Predictors: (Constant), OLeafPotassium  b. Dependent Variable: PLeafPotassium | | | | | | |

| **Coefficients^a^** | | | | | | |
| --- | --- | --- | --- | --- | --- | --- |
| Model | | Unstandardized Coefficients | | Standardized Coefficients | t | Sig. |
|  |  | B | Std. Error | Beta |  |  |
| 1 | (Constant) | .180 | .068 |  | 2.653 | .009 |
|  | OLeafPotassium | .980 | .007 | .995 | 133.403 | .000 |
| a. Dependent Variable: PLeafPotassium | | | | | | |

REGRESSION

/MISSING LISTWISE

/STATISTICS COEFF OUTS R ANOVA

/CRITERIA=PIN(.05) POUT(.10)

/NOORIGIN

/DEPENDENT PRootPotassium

/METHOD=ENTER ORootPotassium.

**Regression**

| **Notes** | | |
| --- | --- | --- |
| Output Created | | 14-Mar-2023 03:06:35 |
| Comments | |  |
| Input | Data | C:\Users\ABAS\Documents\Abbas-Revision.sav |
|  | Active Dataset | DataSet1 |
|  | Filter | <none> |
|  | Weight | <none> |
|  | Split File | <none> |
|  | N of Rows in Working Data File | 192 |
| Missing Value Handling | Definition of Missing | User-defined missing values are treated as missing. |
|  | Cases Used | Statistics are based on cases with no missing values for any variable used. |
| Syntax | | REGRESSION  /MISSING LISTWISE  /STATISTICS COEFF OUTS R ANOVA  /CRITERIA=PIN(.05) POUT(.10)  /NOORIGIN  /DEPENDENT PRootPotassium  /METHOD=ENTER ORootPotassium. |
| Resources | Processor Time | 00 00:00:00.016 |
|  | Elapsed Time | 00 00:00:00.016 |
|  | Memory Required | 1996 bytes |
|  | Additional Memory Required for Residual Plots | 0 bytes |

[DataSet1] C:\Users\ABAS\Documents\Abbas-Revision.sav

| **Variables Entered/Removed^b^** | | | |
| --- | --- | --- | --- |
| Model | Variables Entered | Variables Removed | Method |
| 1 | ORootPotassium | . | Enter |
| a. All requested variables entered.  b. Dependent Variable: PRootPotassium | | | |

| **Model Summary** | | | | |
| --- | --- | --- | --- | --- |
| Model | R | R Square | Adjusted R Square | Std. Error of the Estimate |
| 1 | .979^a^ | .959 | .959 | .59531 |
| a. Predictors: (Constant), ORootPotassium | | | | |

| **ANOVA^b^** | | | | | | |
| --- | --- | --- | --- | --- | --- | --- |
| Model | | Sum of Squares | df | Mean Square | F | Sig. |
| 1 | Regression | 1569.461 | 1 | 1569.461 | 4428.643 | .000^a^ |
|  | Residual | 67.334 | 190 | .354 |  |  |
|  | Total | 1636.795 | 191 |  |  |  |
| a. Predictors: (Constant), ORootPotassium  b. Dependent Variable: PRootPotassium | | | | | | |

| **Coefficients^a^** | | | | | | |
| --- | --- | --- | --- | --- | --- | --- |
| Model | | Unstandardized Coefficients | | Standardized Coefficients | t | Sig. |
|  |  | B | Std. Error | Beta |  |  |
| 1 | (Constant) | .385 | .181 |  | 2.125 | .035 |
|  | ORootPotassium | .962 | .014 | .979 | 66.548 | .000 |
| a. Dependent Variable: PRootPotassium | | | | | | |

REGRESSION

/MISSING LISTWISE

/STATISTICS COEFF OUTS R ANOVA

/CRITERIA=PIN(.05) POUT(.10)

/NOORIGIN

/DEPENDENT PFruitYieldPotassium

/METHOD=ENTER OFruitYieldPotassium.

**Regression**

| **Notes** | | |
| --- | --- | --- |
| Output Created | | 14-Mar-2023 03:06:50 |
| Comments | |  |
| Input | Data | C:\Users\ABAS\Documents\Abbas-Revision.sav |
|  | Active Dataset | DataSet1 |
|  | Filter | <none> |
|  | Weight | <none> |
|  | Split File | <none> |
|  | N of Rows in Working Data File | 192 |
| Missing Value Handling | Definition of Missing | User-defined missing values are treated as missing. |
|  | Cases Used | Statistics are based on cases with no missing values for any variable used. |
| Syntax | | REGRESSION  /MISSING LISTWISE  /STATISTICS COEFF OUTS R ANOVA  /CRITERIA=PIN(.05) POUT(.10)  /NOORIGIN  /DEPENDENT PFruitYieldPotassium  /METHOD=ENTER OFruitYieldPotassium. |
| Resources | Processor Time | 00 00:00:00.015 |
|  | Elapsed Time | 00 00:00:00.018 |
|  | Memory Required | 1996 bytes |
|  | Additional Memory Required for Residual Plots | 0 bytes |

[DataSet1] C:\Users\ABAS\Documents\Abbas-Revision.sav

| **Variables Entered/Removed^b^** | | | |
| --- | --- | --- | --- |
| Model | Variables Entered | Variables Removed | Method |
| 1 | OFruitYieldPotassium | . | Enter |
| a. All requested variables entered.  b. Dependent Variable: PFruitYieldPotassium | | | |

| **Model Summary** | | | | |
| --- | --- | --- | --- | --- |
| Model | R | R Square | Adjusted R Square | Std. Error of the Estimate |
| 1 | .859^a^ | .737 | .736 | .66140 |
| a. Predictors: (Constant), OFruitYieldPotassium | | | | |

| **ANOVA^b^** | | | | | | |
| --- | --- | --- | --- | --- | --- | --- |
| Model | | Sum of Squares | df | Mean Square | F | Sig. |
| 1 | Regression | 233.147 | 1 | 233.147 | 532.976 | .000^a^ |
|  | Residual | 83.114 | 190 | .437 |  |  |
|  | Total | 316.261 | 191 |  |  |  |
| a. Predictors: (Constant), OFruitYieldPotassium  b. Dependent Variable: PFruitYieldPotassium | | | | | | |

| **Coefficients^a^** | | | | | | |
| --- | --- | --- | --- | --- | --- | --- |
| Model | | Unstandardized Coefficients | | Standardized Coefficients | t | Sig. |
|  |  | B | Std. Error | Beta |  |  |
| 1 | (Constant) | .858 | .140 |  | 6.132 | .000 |
|  | OFruitYieldPotassium | .839 | .036 | .859 | 23.086 | .000 |
| a. Dependent Variable: PFruitYieldPotassium | | | | | | |
