# Supplementary material for: Prediction models of macro-nutrient content in plant organs of Cucumis melo in response to soil elements using support vector regression
Source: PeerJ. 2023 Oct 2;11:e15417. doi: 10.7717/peerj.15417 (PMC10552743; doi:10.7717/peerj.15417)
Supplement: Supplemental Information 15 — Results of the performance function and the statistical description of observed and predicted values of nitrogen, phosphorus, and potassium in seeds, fruits, leaves, and roots were calculated with Matlab V7.1 software according to the methodology described in Methods. The final data is presented in Tables. [file peerj-11-15417-s015.docx]

DESCRIPTIVES VARIABLES=N1O N2O N3O N4O

/STATISTICS=MEAN STDDEV MIN MAX SEMEAN.

**Descriptives**

| **Notes** | | |
| --- | --- | --- |
| Output Created | | 13-Jan-2021 05:22:09 |
| Comments | |  |
| Input | Active Dataset | DataSet0 |
|  | Filter | <none> |
|  | Weight | <none> |
|  | Split File | <none> |
|  | N of Rows in Working Data File | 144 |
| Missing Value Handling | Definition of Missing | User defined missing values are treated as missing. |
|  | Cases Used | All non-missing data are used. |
| Syntax | | DESCRIPTIVES VARIABLES=N1O N2O N3O N4O  /STATISTICS=MEAN STDDEV MIN MAX SEMEAN. |
| Resources | Processor Time | 00 00:00:00.000 |
|  | Elapsed Time | 00 00:00:00.000 |

[DataSet0]

| **Descriptive Statistics** | | | | | | |
| --- | --- | --- | --- | --- | --- | --- |
|  | N | Minimum | Maximum | Mean | | Std. Deviation |
|  | Statistic | Statistic | Statistic | Statistic | Std. Error | Statistic |
| N1O | 144 | 1.44 | 2.94 | 2.1514 | .03395 | .40735 |
| N2O | 144 | .79 | 1.99 | 1.2337 | .02322 | .27860 |
| N3O | 144 | 2.17 | 5.48 | 3.1826 | .06664 | .79965 |
| N4O | 144 | .71 | 1.88 | 1.0436 | .02629 | .31550 |
| Valid N (listwise) | 144 |  |  |  |  |  |

DESCRIPTIVES VARIABLES=N1P N2P N3P N4P

/STATISTICS=MEAN STDDEV MIN MAX SEMEAN.

**Descriptives**

| **Notes** | | |
| --- | --- | --- |
| Output Created | | 13-Jan-2021 05:22:28 |
| Comments | |  |
| Input | Active Dataset | DataSet0 |
|  | Filter | <none> |
|  | Weight | <none> |
|  | Split File | <none> |
|  | N of Rows in Working Data File | 144 |
| Missing Value Handling | Definition of Missing | User defined missing values are treated as missing. |
|  | Cases Used | All non-missing data are used. |
| Syntax | | DESCRIPTIVES VARIABLES=N1P N2P N3P N4P  /STATISTICS=MEAN STDDEV MIN MAX SEMEAN. |
| Resources | Processor Time | 00 00:00:00.000 |
|  | Elapsed Time | 00 00:00:00.000 |

[DataSet0]

| **Descriptive Statistics** | | | | | | |
| --- | --- | --- | --- | --- | --- | --- |
|  | N | Minimum | Maximum | Mean | | Std. Deviation |
|  | Statistic | Statistic | Statistic | Statistic | Std. Error | Statistic |
| N1P | 144 | 1.39 | 2.83 | 2.1369 | .02807 | .33686 |
| N2P | 144 | .68 | 1.94 | 1.2282 | .02055 | .24666 |
| N3P | 144 | 2.13 | 5.50 | 3.2155 | .06278 | .75336 |
| N4P | 144 | .66 | 1.85 | 1.0257 | .02233 | .26791 |
| Valid N (listwise) | 144 |  |  |  |  |  |

DESCRIPTIVES VARIABLES=P1O P2O P3O P4O

/STATISTICS=MEAN STDDEV MIN MAX SEMEAN.

**Descriptives**

| **Notes** | | |
| --- | --- | --- |
| Output Created | | 13-Jan-2021 05:22:47 |
| Comments | |  |
| Input | Active Dataset | DataSet0 |
|  | Filter | <none> |
|  | Weight | <none> |
|  | Split File | <none> |
|  | N of Rows in Working Data File | 144 |
| Missing Value Handling | Definition of Missing | User defined missing values are treated as missing. |
|  | Cases Used | All non-missing data are used. |
| Syntax | | DESCRIPTIVES VARIABLES=P1O P2O P3O P4O  /STATISTICS=MEAN STDDEV MIN MAX SEMEAN. |
| Resources | Processor Time | 00 00:00:00.000 |
|  | Elapsed Time | 00 00:00:00.000 |

[DataSet0]

| **Descriptive Statistics** | | | | | | |
| --- | --- | --- | --- | --- | --- | --- |
|  | N | Minimum | Maximum | Mean | | Std. Deviation |
|  | Statistic | Statistic | Statistic | Statistic | Std. Error | Statistic |
| P1O | 144 | 15.21 | 46.80 | 23.9160 | .48958 | 5.87500 |
| P2O | 144 | 10.29 | 40.88 | 22.9683 | .53190 | 6.38281 |
| P3O | 144 | 14.60 | 34.39 | 24.0039 | .40157 | 4.81885 |
| P4O | 144 | 9.72 | 56.55 | 21.6840 | .65754 | 7.89048 |
| Valid N (listwise) | 144 |  |  |  |  |  |

DESCRIPTIVES VARIABLES=P1P P2P P3P P4P

/STATISTICS=MEAN STDDEV MIN MAX SEMEAN.

**Descriptives**

| **Notes** | | |
| --- | --- | --- |
| Output Created | | 13-Jan-2021 05:23:14 |
| Comments | |  |
| Input | Active Dataset | DataSet0 |
|  | Filter | <none> |
|  | Weight | <none> |
|  | Split File | <none> |
|  | N of Rows in Working Data File | 144 |
| Missing Value Handling | Definition of Missing | User defined missing values are treated as missing. |
|  | Cases Used | All non-missing data are used. |
| Syntax | | DESCRIPTIVES VARIABLES=P1P P2P P3P P4P  /STATISTICS=MEAN STDDEV MIN MAX SEMEAN. |
| Resources | Processor Time | 00 00:00:00.000 |
|  | Elapsed Time | 00 00:00:00.000 |

[DataSet0]

| **Descriptive Statistics** | | | | | | |
| --- | --- | --- | --- | --- | --- | --- |
|  | N | Minimum | Maximum | Mean | | Std. Deviation |
|  | Statistic | Statistic | Statistic | Statistic | Std. Error | Statistic |
| P1P | 144 | 15.18 | 44.55 | 23.9158 | .48521 | 5.82251 |
| P2P | 144 | 10.34 | 40.83 | 22.9553 | .53130 | 6.37566 |
| P3P | 144 | 14.55 | 32.85 | 23.9667 | .39858 | 4.78299 |
| P4P | 144 | 9.77 | 55.49 | 21.7057 | .65140 | 7.81685 |
| Valid N (listwise) | 144 |  |  |  |  |  |

DESCRIPTIVES VARIABLES=K1O K2O K3O K4O

/STATISTICS=MEAN STDDEV MIN MAX SEMEAN.

**Descriptives**

| **Notes** | | |
| --- | --- | --- |
| Output Created | | 13-Jan-2021 05:23:38 |
| Comments | |  |
| Input | Active Dataset | DataSet0 |
|  | Filter | <none> |
|  | Weight | <none> |
|  | Split File | <none> |
|  | N of Rows in Working Data File | 144 |
| Missing Value Handling | Definition of Missing | User defined missing values are treated as missing. |
|  | Cases Used | All non-missing data are used. |
| Syntax | | DESCRIPTIVES VARIABLES=K1O K2O K3O K4O  /STATISTICS=MEAN STDDEV MIN MAX SEMEAN. |
| Resources | Processor Time | 00 00:00:00.016 |
|  | Elapsed Time | 00 00:00:00.014 |

[DataSet0]

| **Descriptive Statistics** | | | | | | |
| --- | --- | --- | --- | --- | --- | --- |
|  | N | Minimum | Maximum | Mean | | Std. Deviation |
|  | Statistic | Statistic | Statistic | Statistic | Std. Error | Statistic |
| K1O | 144 | 9.62 | 22.93 | 14.3452 | .22387 | 2.68643 |
| K2O | 144 | 12.23 | 22.84 | 16.8314 | .21168 | 2.54017 |
| K3O | 144 | 5.75 | 13.80 | 9.0990 | .15277 | 1.83319 |
| K4O | 144 | 5.42 | 19.02 | 11.9165 | .26522 | 3.18262 |
| Valid N (listwise) | 144 |  |  |  |  |  |

DESCRIPTIVES VARIABLES=K1P K2P K3P K4P

/STATISTICS=MEAN STDDEV MIN MAX SEMEAN.

**Descriptives**

| **Notes** | | |
| --- | --- | --- |
| Output Created | | 13-Jan-2021 05:23:55 |
| Comments | |  |
| Input | Active Dataset | DataSet0 |
|  | Filter | <none> |
|  | Weight | <none> |
|  | Split File | <none> |
|  | N of Rows in Working Data File | 144 |
| Missing Value Handling | Definition of Missing | User defined missing values are treated as missing. |
|  | Cases Used | All non-missing data are used. |
| Syntax | | DESCRIPTIVES VARIABLES=K1P K2P K3P K4P  /STATISTICS=MEAN STDDEV MIN MAX SEMEAN. |
| Resources | Processor Time | 00 00:00:00.000 |
|  | Elapsed Time | 00 00:00:00.000 |

[DataSet0]

| **Descriptive Statistics** | | | | | | |
| --- | --- | --- | --- | --- | --- | --- |
|  | N | Minimum | Maximum | Mean | | Std. Deviation |
|  | Statistic | Statistic | Statistic | Statistic | Std. Error | Statistic |
| K1P | 144 | 9.82 | 22.73 | 14.2749 | .20284 | 2.43405 |
| K2P | 144 | 12.43 | 22.62 | 16.7845 | .20056 | 2.40673 |
| K3P | 144 | 5.80 | 13.75 | 9.0962 | .14937 | 1.79242 |
| K4P | 144 | 5.47 | 18.89 | 11.8062 | .25884 | 3.10607 |
| Valid N (listwise) | 144 |  |  |  |  |  |

REGRESSION

/DESCRIPTIVES MEAN STDDEV CORR SIG N

/MISSING LISTWISE

/STATISTICS COEFF OUTS R ANOVA CHANGE

/CRITERIA=PIN(.05) POUT(.10)

/NOORIGIN

/DEPENDENT N1O

/METHOD=ENTER N1P

/RESIDUALS DURBIN.

**Regression**

| **Notes** | | |
| --- | --- | --- |
| Output Created | | 13-Jan-2021 05:26:55 |
| Comments | |  |
| Input | Active Dataset | DataSet0 |
|  | Filter | <none> |
|  | Weight | <none> |
|  | Split File | <none> |
|  | N of Rows in Working Data File | 144 |
| Missing Value Handling | Definition of Missing | User-defined missing values are treated as missing. |
|  | Cases Used | Statistics are based on cases with no missing values for any variable used. |
| Syntax | | REGRESSION  /DESCRIPTIVES MEAN STDDEV CORR SIG N  /MISSING LISTWISE  /STATISTICS COEFF OUTS R ANOVA CHANGE  /CRITERIA=PIN(.05) POUT(.10)  /NOORIGIN  /DEPENDENT N1O  /METHOD=ENTER N1P  /RESIDUALS DURBIN. |
| Resources | Processor Time | 00 00:00:00.032 |
|  | Elapsed Time | 00 00:00:00.344 |
|  | Memory Required | 1796 bytes |
|  | Additional Memory Required for Residual Plots | 0 bytes |

[DataSet0]

| **Descriptive Statistics** | | | |
| --- | --- | --- | --- |
|  | Mean | Std. Deviation | N |
| N1O | 2.1514 | .40735 | 144 |
| N1P | 2.1369 | .33686 | 144 |

| **Correlations** | | | |
| --- | --- | --- | --- |
|  | | N1O | N1P |
| Pearson Correlation | N1O | 1.000 | .835 |
|  | N1P | .835 | 1.000 |
| Sig. (1-tailed) | N1O | . | .000 |
|  | N1P | .000 | . |
| N | N1O | 144 | 144 |
|  | N1P | 144 | 144 |

| **Variables Entered/Removed^b^** | | | |
| --- | --- | --- | --- |
| Model | Variables Entered | Variables Removed | Method |
| 1 | N1P^a^ | . | Enter |
| a. All requested variables entered.  b. Dependent Variable: N1O | | | |

| **Model Summary^b^** | | | | |
| --- | --- | --- | --- | --- |
| Model | R | R Square | Adjusted R Square | Std. Error of the Estimate |
| 1 | .835^a^ | .697 | .695 | .22513 |

| **Model Summary^b^** | | | | | | |
| --- | --- | --- | --- | --- | --- | --- |
| Model | Change Statistics | | | | | Durbin-Watson |
|  | R Square Change | F Change | df1 | df2 | Sig. F Change |  |
| 1 | .697 | 326.164 | 1 | 142 | .000 | 1.725 |

|  |
| --- |
| a. Predictors: (Constant), N1P  b. Dependent Variable: N1O |

| **ANOVA^b^** | | | | | | |
| --- | --- | --- | --- | --- | --- | --- |
| Model | | Sum of Squares | df | Mean Square | F | Sig. |
| 1 | Regression | 16.532 | 1 | 16.532 | 326.164 | .000^a^ |
|  | Residual | 7.197 | 142 | .051 |  |  |
|  | Total | 23.729 | 143 |  |  |  |
| a. Predictors: (Constant), N1P  b. Dependent Variable: N1O | | | | | | |

| **Coefficients^a^** | | | | | | |
| --- | --- | --- | --- | --- | --- | --- |
| Model | | Unstandardized Coefficients | | Standardized Coefficients | t | Sig. |
|  |  | B | Std. Error | Beta |  |  |
| 1 | (Constant) | -.005 | .121 |  | -.045 | .964 |
|  | N1P | 1.009 | .056 | .835 | 18.060 | .000 |
| a. Dependent Variable: N1O | | | | | | |

| **Residuals Statistics^a^** | | | | | |
| --- | --- | --- | --- | --- | --- |
|  | Minimum | Maximum | Mean | Std. Deviation | N |
| Predicted Value | 1.3966 | 2.8493 | 2.1514 | .34001 | 144 |
| Residual | -.71536 | .75359 | .00000 | .22435 | 144 |
| Std. Predicted Value | -2.220 | 2.053 | .000 | 1.000 | 144 |
| Std. Residual | -3.177 | 3.347 | .000 | .996 | 144 |
| a. Dependent Variable: N1O | | | | | |

REGRESSION

/DESCRIPTIVES MEAN STDDEV CORR SIG N

/MISSING LISTWISE

/STATISTICS COEFF OUTS R ANOVA CHANGE

/CRITERIA=PIN(.05) POUT(.10)

/NOORIGIN

/DEPENDENT N2O

/METHOD=ENTER N2P

/RESIDUALS DURBIN.

**Regression**

| **Notes** | | |
| --- | --- | --- |
| Output Created | | 13-Jan-2021 05:29:26 |
| Comments | |  |
| Input | Active Dataset | DataSet0 |
|  | Filter | <none> |
|  | Weight | <none> |
|  | Split File | <none> |
|  | N of Rows in Working Data File | 144 |
| Missing Value Handling | Definition of Missing | User-defined missing values are treated as missing. |
|  | Cases Used | Statistics are based on cases with no missing values for any variable used. |
| Syntax | | REGRESSION  /DESCRIPTIVES MEAN STDDEV CORR SIG N  /MISSING LISTWISE  /STATISTICS COEFF OUTS R ANOVA CHANGE  /CRITERIA=PIN(.05) POUT(.10)  /NOORIGIN  /DEPENDENT N2O  /METHOD=ENTER N2P  /RESIDUALS DURBIN. |
| Resources | Processor Time | 00 00:00:00.031 |
|  | Elapsed Time | 00 00:00:00.110 |
|  | Memory Required | 1796 bytes |
|  | Additional Memory Required for Residual Plots | 0 bytes |

[DataSet0]

| **Descriptive Statistics** | | | |
| --- | --- | --- | --- |
|  | Mean | Std. Deviation | N |
| N2O | 1.2337 | .27860 | 144 |
| N2P | 1.2282 | .24666 | 144 |

| **Correlations** | | | |
| --- | --- | --- | --- |
|  | | N2O | N2P |
| Pearson Correlation | N2O | 1.000 | .898 |
|  | N2P | .898 | 1.000 |
| Sig. (1-tailed) | N2O | . | .000 |
|  | N2P | .000 | . |
| N | N2O | 144 | 144 |
|  | N2P | 144 | 144 |

| **Variables Entered/Removed^b^** | | | |
| --- | --- | --- | --- |
| Model | Variables Entered | Variables Removed | Method |
| 1 | N2P^a^ | . | Enter |
| a. All requested variables entered.  b. Dependent Variable: N2O | | | |

| **Model Summary^b^** | | | | |
| --- | --- | --- | --- | --- |
| Model | R | R Square | Adjusted R Square | Std. Error of the Estimate |
| 1 | .898^a^ | .807 | .805 | .12291 |

| **Model Summary^b^** | | | | | | |
| --- | --- | --- | --- | --- | --- | --- |
| Model | Change Statistics | | | | | Durbin-Watson |
|  | R Square Change | F Change | df1 | df2 | Sig. F Change |  |
| 1 | .807 | 592.695 | 1 | 142 | .000 | 2.029 |

|  |
| --- |
| a. Predictors: (Constant), N2P  b. Dependent Variable: N2O |

| **ANOVA^b^** | | | | | | |
| --- | --- | --- | --- | --- | --- | --- |
| Model | | Sum of Squares | df | Mean Square | F | Sig. |
| 1 | Regression | 8.954 | 1 | 8.954 | 592.695 | .000^a^ |
|  | Residual | 2.145 | 142 | .015 |  |  |
|  | Total | 11.100 | 143 |  |  |  |
| a. Predictors: (Constant), N2P  b. Dependent Variable: N2O | | | | | | |

| **Coefficients^a^** | | | | | | |
| --- | --- | --- | --- | --- | --- | --- |
| Model | | Unstandardized Coefficients | | Standardized Coefficients | t | Sig. |
|  |  | B | Std. Error | Beta |  |  |
| 1 | (Constant) | -.012 | .052 |  | -.237 | .813 |
|  | N2P | 1.015 | .042 | .898 | 24.345 | .000 |
| a. Dependent Variable: N2O | | | | | | |

| **Residuals Statistics^a^** | | | | | |
| --- | --- | --- | --- | --- | --- |
|  | Minimum | Maximum | Mean | Std. Deviation | N |
| Predicted Value | .6750 | 1.9570 | 1.2337 | .25023 | 144 |
| Residual | -.39368 | .57887 | .00000 | .12248 | 144 |
| Std. Predicted Value | -2.232 | 2.891 | .000 | 1.000 | 144 |
| Std. Residual | -3.203 | 4.710 | .000 | .996 | 144 |
| a. Dependent Variable: N2O | | | | | |

REGRESSION

/DESCRIPTIVES MEAN STDDEV CORR SIG N

/MISSING LISTWISE

/STATISTICS COEFF OUTS R ANOVA CHANGE

/CRITERIA=PIN(.05) POUT(.10)

/NOORIGIN

/DEPENDENT N3O

/METHOD=ENTER N3P

/RESIDUALS DURBIN.

**Regression**

| **Notes** | | |
| --- | --- | --- |
| Output Created | | 13-Jan-2021 05:30:29 |
| Comments | |  |
| Input | Active Dataset | DataSet0 |
|  | Filter | <none> |
|  | Weight | <none> |
|  | Split File | <none> |
|  | N of Rows in Working Data File | 144 |
| Missing Value Handling | Definition of Missing | User-defined missing values are treated as missing. |
|  | Cases Used | Statistics are based on cases with no missing values for any variable used. |
| Syntax | | REGRESSION  /DESCRIPTIVES MEAN STDDEV CORR SIG N  /MISSING LISTWISE  /STATISTICS COEFF OUTS R ANOVA CHANGE  /CRITERIA=PIN(.05) POUT(.10)  /NOORIGIN  /DEPENDENT N3O  /METHOD=ENTER N3P  /RESIDUALS DURBIN. |
| Resources | Processor Time | 00 00:00:00.016 |
|  | Elapsed Time | 00 00:00:00.062 |
|  | Memory Required | 1796 bytes |
|  | Additional Memory Required for Residual Plots | 0 bytes |

[DataSet0]

| **Descriptive Statistics** | | | |
| --- | --- | --- | --- |
|  | Mean | Std. Deviation | N |
| N3O | 3.1826 | .79965 | 144 |
| N3P | 3.2155 | .75336 | 144 |

| **Correlations** | | | |
| --- | --- | --- | --- |
|  | | N3O | N3P |
| Pearson Correlation | N3O | 1.000 | .912 |
|  | N3P | .912 | 1.000 |
| Sig. (1-tailed) | N3O | . | .000 |
|  | N3P | .000 | . |
| N | N3O | 144 | 144 |
|  | N3P | 144 | 144 |

| **Variables Entered/Removed^b^** | | | |
| --- | --- | --- | --- |
| Model | Variables Entered | Variables Removed | Method |
| 1 | N3P^a^ | . | Enter |
| a. All requested variables entered.  b. Dependent Variable: N3O | | | |

| **Model Summary^b^** | | | | |
| --- | --- | --- | --- | --- |
| Model | R | R Square | Adjusted R Square | Std. Error of the Estimate |
| 1 | .912^a^ | .832 | .831 | .32891 |

| **Model Summary^b^** | | | | | | |
| --- | --- | --- | --- | --- | --- | --- |
| Model | Change Statistics | | | | | Durbin-Watson |
|  | R Square Change | F Change | df1 | df2 | Sig. F Change |  |
| 1 | .832 | 703.258 | 1 | 142 | .000 | 1.885 |

|  |
| --- |
| a. Predictors: (Constant), N3P  b. Dependent Variable: N3O |

| **ANOVA^b^** | | | | | | |
| --- | --- | --- | --- | --- | --- | --- |
| Model | | Sum of Squares | df | Mean Square | F | Sig. |
| 1 | Regression | 76.078 | 1 | 76.078 | 703.258 | .000^a^ |
|  | Residual | 15.361 | 142 | .108 |  |  |
|  | Total | 91.439 | 143 |  |  |  |
| a. Predictors: (Constant), N3P  b. Dependent Variable: N3O | | | | | | |

| **Coefficients^a^** | | | | | | |
| --- | --- | --- | --- | --- | --- | --- |
| Model | | Unstandardized Coefficients | | Standardized Coefficients | t | Sig. |
|  |  | B | Std. Error | Beta |  |  |
| 1 | (Constant) | .069 | .121 |  | .575 | .566 |
|  | N3P | .968 | .037 | .912 | 26.519 | .000 |
| a. Dependent Variable: N3O | | | | | | |

| **Residuals Statistics^a^** | | | | | |
| --- | --- | --- | --- | --- | --- |
|  | Minimum | Maximum | Mean | Std. Deviation | N |
| Predicted Value | 2.1309 | 5.3986 | 3.1826 | .72939 | 144 |
| Residual | -2.55071 | .98789 | .00000 | .32775 | 144 |
| Std. Predicted Value | -1.442 | 3.038 | .000 | 1.000 | 144 |
| Std. Residual | -7.755 | 3.004 | .000 | .996 | 144 |
| a. Dependent Variable: N3O | | | | | |

REGRESSION

/DESCRIPTIVES MEAN STDDEV CORR SIG N

/MISSING LISTWISE

/STATISTICS COEFF OUTS R ANOVA CHANGE

/CRITERIA=PIN(.05) POUT(.10)

/NOORIGIN

/DEPENDENT N4O

/METHOD=ENTER N4P

/RESIDUALS DURBIN.

**Regression**

| **Notes** | | |
| --- | --- | --- |
| Output Created | | 13-Jan-2021 05:35:49 |
| Comments | |  |
| Input | Active Dataset | DataSet0 |
|  | Filter | <none> |
|  | Weight | <none> |
|  | Split File | <none> |
|  | N of Rows in Working Data File | 144 |
| Missing Value Handling | Definition of Missing | User-defined missing values are treated as missing. |
|  | Cases Used | Statistics are based on cases with no missing values for any variable used. |
| Syntax | | REGRESSION  /DESCRIPTIVES MEAN STDDEV CORR SIG N  /MISSING LISTWISE  /STATISTICS COEFF OUTS R ANOVA CHANGE  /CRITERIA=PIN(.05) POUT(.10)  /NOORIGIN  /DEPENDENT N4O  /METHOD=ENTER N4P  /RESIDUALS DURBIN. |
| Resources | Processor Time | 00 00:00:00.016 |
|  | Elapsed Time | 00 00:00:00.032 |
|  | Memory Required | 1796 bytes |
|  | Additional Memory Required for Residual Plots | 0 bytes |

[DataSet0]

| **Descriptive Statistics** | | | |
| --- | --- | --- | --- |
|  | Mean | Std. Deviation | N |
| N4O | 1.0436 | .31550 | 144 |
| N4P | 1.0257 | .26791 | 144 |

| **Correlations** | | | |
| --- | --- | --- | --- |
|  | | N4O | N4P |
| Pearson Correlation | N4O | 1.000 | .736 |
|  | N4P | .736 | 1.000 |
| Sig. (1-tailed) | N4O | . | .000 |
|  | N4P | .000 | . |
| N | N4O | 144 | 144 |
|  | N4P | 144 | 144 |

| **Variables Entered/Removed^b^** | | | |
| --- | --- | --- | --- |
| Model | Variables Entered | Variables Removed | Method |
| 1 | N4P^a^ | . | Enter |
| a. All requested variables entered.  b. Dependent Variable: N4O | | | |

| **Model Summary^b^** | | | | |
| --- | --- | --- | --- | --- |
| Model | R | R Square | Adjusted R Square | Std. Error of the Estimate |
| 1 | .736^a^ | .542 | .539 | .21421 |

| **Model Summary^b^** | | | | | | |
| --- | --- | --- | --- | --- | --- | --- |
| Model | Change Statistics | | | | | Durbin-Watson |
|  | R Square Change | F Change | df1 | df2 | Sig. F Change |  |
| 1 | .542 | 168.227 | 1 | 142 | .000 | 1.935 |

|  |
| --- |
| a. Predictors: (Constant), N4P  b. Dependent Variable: N4O |

| **ANOVA^b^** | | | | | | |
| --- | --- | --- | --- | --- | --- | --- |
| Model | | Sum of Squares | df | Mean Square | F | Sig. |
| 1 | Regression | 7.719 | 1 | 7.719 | 168.227 | .000^a^ |
|  | Residual | 6.516 | 142 | .046 |  |  |
|  | Total | 14.235 | 143 |  |  |  |
| a. Predictors: (Constant), N4P  b. Dependent Variable: N4O | | | | | | |

| **Coefficients^a^** | | | | | | |
| --- | --- | --- | --- | --- | --- | --- |
| Model | | Unstandardized Coefficients | | Standardized Coefficients | t | Sig. |
|  |  | B | Std. Error | Beta |  |  |
| 1 | (Constant) | .154 | .071 |  | 2.176 | .031 |
|  | N4P | .867 | .067 | .736 | 12.970 | .000 |
| a. Dependent Variable: N4O | | | | | | |

| **Residuals Statistics^a^** | | | | | |
| --- | --- | --- | --- | --- | --- |
|  | Minimum | Maximum | Mean | Std. Deviation | N |
| Predicted Value | .7257 | 1.7599 | 1.0436 | .23233 | 144 |
| Residual | -.75760 | .58482 | .00000 | .21346 | 144 |
| Std. Predicted Value | -1.368 | 3.083 | .000 | 1.000 | 144 |
| Std. Residual | -3.537 | 2.730 | .000 | .996 | 144 |
| a. Dependent Variable: N4O | | | | | |

REGRESSION

/DESCRIPTIVES MEAN STDDEV CORR SIG N

/MISSING LISTWISE

/STATISTICS COEFF OUTS R ANOVA CHANGE

/CRITERIA=PIN(.05) POUT(.10)

/NOORIGIN

/DEPENDENT P1O

/METHOD=ENTER P1P

/RESIDUALS DURBIN.

**Regression**

| **Notes** | | |
| --- | --- | --- |
| Output Created | | 13-Jan-2021 05:36:19 |
| Comments | |  |
| Input | Active Dataset | DataSet0 |
|  | Filter | <none> |
|  | Weight | <none> |
|  | Split File | <none> |
|  | N of Rows in Working Data File | 144 |
| Missing Value Handling | Definition of Missing | User-defined missing values are treated as missing. |
|  | Cases Used | Statistics are based on cases with no missing values for any variable used. |
| Syntax | | REGRESSION  /DESCRIPTIVES MEAN STDDEV CORR SIG N  /MISSING LISTWISE  /STATISTICS COEFF OUTS R ANOVA CHANGE  /CRITERIA=PIN(.05) POUT(.10)  /NOORIGIN  /DEPENDENT P1O  /METHOD=ENTER P1P  /RESIDUALS DURBIN. |
| Resources | Processor Time | 00 00:00:00.016 |
|  | Elapsed Time | 00 00:00:00.111 |
|  | Memory Required | 1796 bytes |
|  | Additional Memory Required for Residual Plots | 0 bytes |

[DataSet0]

| **Descriptive Statistics** | | | |
| --- | --- | --- | --- |
|  | Mean | Std. Deviation | N |
| P1O | 23.9160 | 5.87500 | 144 |
| P1P | 23.9158 | 5.82251 | 144 |

| **Correlations** | | | |
| --- | --- | --- | --- |
|  | | P1O | P1P |
| Pearson Correlation | P1O | 1.000 | .998 |
|  | P1P | .998 | 1.000 |
| Sig. (1-tailed) | P1O | . | .000 |
|  | P1P | .000 | . |
| N | P1O | 144 | 144 |
|  | P1P | 144 | 144 |

| **Variables Entered/Removed^b^** | | | |
| --- | --- | --- | --- |
| Model | Variables Entered | Variables Removed | Method |
| 1 | P1P^a^ | . | Enter |
| a. All requested variables entered.  b. Dependent Variable: P1O | | | |

| **Model Summary^b^** | | | | |
| --- | --- | --- | --- | --- |
| Model | R | R Square | Adjusted R Square | Std. Error of the Estimate |
| 1 | .998^a^ | .997 | .997 | .33324 |

| **Model Summary^b^** | | | | | | |
| --- | --- | --- | --- | --- | --- | --- |
| Model | Change Statistics | | | | | Durbin-Watson |
|  | R Square Change | F Change | df1 | df2 | Sig. F Change |  |
| 1 | .997 | 44304.374 | 1 | 142 | .000 | 2.029 |

|  |
| --- |
| a. Predictors: (Constant), P1P  b. Dependent Variable: P1O |

| **ANOVA^b^** | | | | | | |
| --- | --- | --- | --- | --- | --- | --- |
| Model | | Sum of Squares | df | Mean Square | F | Sig. |
| 1 | Regression | 4919.959 | 1 | 4919.959 | 44304.374 | .000^a^ |
|  | Residual | 15.769 | 142 | .111 |  |  |
|  | Total | 4935.728 | 143 |  |  |  |
| a. Predictors: (Constant), P1P  b. Dependent Variable: P1O | | | | | | |

| **Coefficients^a^** | | | | | | |
| --- | --- | --- | --- | --- | --- | --- |
| Model | | Unstandardized Coefficients | | Standardized Coefficients | t | Sig. |
|  |  | B | Std. Error | Beta |  |  |
| 1 | (Constant) | -.177 | .118 |  | -1.501 | .136 |
|  | P1P | 1.007 | .005 | .998 | 210.486 | .000 |
| a. Dependent Variable: P1O | | | | | | |

| **Residuals Statistics^a^** | | | | | |
| --- | --- | --- | --- | --- | --- |
|  | Minimum | Maximum | Mean | Std. Deviation | N |
| Predicted Value | 15.1154 | 44.7048 | 23.9160 | 5.86560 | 144 |
| Residual | -1.80720 | 2.09516 | .00000 | .33207 | 144 |
| Std. Predicted Value | -1.500 | 3.544 | .000 | 1.000 | 144 |
| Std. Residual | -5.423 | 6.287 | .000 | .996 | 144 |
| a. Dependent Variable: P1O | | | | | |

REGRESSION

/DESCRIPTIVES MEAN STDDEV CORR SIG N

/MISSING LISTWISE

/STATISTICS COEFF OUTS R ANOVA CHANGE

/CRITERIA=PIN(.05) POUT(.10)

/NOORIGIN

/DEPENDENT P2O

/METHOD=ENTER P2P

/RESIDUALS DURBIN.

**Regression**

| **Notes** | | |
| --- | --- | --- |
| Output Created | | 13-Jan-2021 05:38:15 |
| Comments | |  |
| Input | Active Dataset | DataSet0 |
|  | Filter | <none> |
|  | Weight | <none> |
|  | Split File | <none> |
|  | N of Rows in Working Data File | 144 |
| Missing Value Handling | Definition of Missing | User-defined missing values are treated as missing. |
|  | Cases Used | Statistics are based on cases with no missing values for any variable used. |
| Syntax | | REGRESSION  /DESCRIPTIVES MEAN STDDEV CORR SIG N  /MISSING LISTWISE  /STATISTICS COEFF OUTS R ANOVA CHANGE  /CRITERIA=PIN(.05) POUT(.10)  /NOORIGIN  /DEPENDENT P2O  /METHOD=ENTER P2P  /RESIDUALS DURBIN. |
| Resources | Processor Time | 00 00:00:00.015 |
|  | Elapsed Time | 00 00:00:00.031 |
|  | Memory Required | 1796 bytes |
|  | Additional Memory Required for Residual Plots | 0 bytes |

[DataSet0]

| **Descriptive Statistics** | | | |
| --- | --- | --- | --- |
|  | Mean | Std. Deviation | N |
| P2O | 22.9683 | 6.38281 | 144 |
| P2P | 22.9553 | 6.37566 | 144 |

| **Correlations** | | | |
| --- | --- | --- | --- |
|  | | P2O | P2P |
| Pearson Correlation | P2O | 1.000 | .999 |
|  | P2P | .999 | 1.000 |
| Sig. (1-tailed) | P2O | . | .000 |
|  | P2P | .000 | . |
| N | P2O | 144 | 144 |
|  | P2P | 144 | 144 |

| **Variables Entered/Removed^b^** | | | |
| --- | --- | --- | --- |
| Model | Variables Entered | Variables Removed | Method |
| 1 | P2P^a^ | . | Enter |
| a. All requested variables entered.  b. Dependent Variable: P2O | | | |

| **Model Summary^b^** | | | | |
| --- | --- | --- | --- | --- |
| Model | R | R Square | Adjusted R Square | Std. Error of the Estimate |
| 1 | .999^a^ | .999 | .999 | .22893 |

| **Model Summary^b^** | | | | | | |
| --- | --- | --- | --- | --- | --- | --- |
| Model | Change Statistics | | | | | Durbin-Watson |
|  | R Square Change | F Change | df1 | df2 | Sig. F Change |  |
| 1 | .999 | 111014.666 | 1 | 142 | .000 | 1.937 |

|  |
| --- |
| a. Predictors: (Constant), P2P  b. Dependent Variable: P2O |

| **ANOVA^b^** | | | | | | |
| --- | --- | --- | --- | --- | --- | --- |
| Model | | Sum of Squares | df | Mean Square | F | Sig. |
| 1 | Regression | 5818.413 | 1 | 5818.413 | 111014.666 | .000^a^ |
|  | Residual | 7.442 | 142 | .052 |  |  |
|  | Total | 5825.856 | 143 |  |  |  |
| a. Predictors: (Constant), P2P  b. Dependent Variable: P2O | | | | | | |

| **Coefficients^a^** | | | | | | |
| --- | --- | --- | --- | --- | --- | --- |
| Model | | Unstandardized Coefficients | | Standardized Coefficients | t | Sig. |
|  |  | B | Std. Error | Beta |  |  |
| 1 | (Constant) | .002 | .072 |  | .027 | .978 |
|  | P2P | 1.000 | .003 | .999 | 333.189 | .000 |
| a. Dependent Variable: P2O | | | | | | |

| **Residuals Statistics^a^** | | | | | |
| --- | --- | --- | --- | --- | --- |
|  | Minimum | Maximum | Mean | Std. Deviation | N |
| Predicted Value | 10.3472 | 40.8515 | 22.9683 | 6.37873 | 144 |
| Residual | -1.16517 | 2.39007 | .00000 | .22813 | 144 |
| Std. Predicted Value | -1.979 | 2.804 | .000 | 1.000 | 144 |
| Std. Residual | -5.090 | 10.440 | .000 | .996 | 144 |
| a. Dependent Variable: P2O | | | | | |

REGRESSION

/DESCRIPTIVES MEAN STDDEV CORR SIG N

/MISSING LISTWISE

/STATISTICS COEFF OUTS R ANOVA CHANGE

/CRITERIA=PIN(.05) POUT(.10)

/NOORIGIN

/DEPENDENT P3O

/METHOD=ENTER P3P

/RESIDUALS DURBIN.

**Regression**

| **Notes** | | |
| --- | --- | --- |
| Output Created | | 13-Jan-2021 05:38:34 |
| Comments | |  |
| Input | Active Dataset | DataSet0 |
|  | Filter | <none> |
|  | Weight | <none> |
|  | Split File | <none> |
|  | N of Rows in Working Data File | 144 |
| Missing Value Handling | Definition of Missing | User-defined missing values are treated as missing. |
|  | Cases Used | Statistics are based on cases with no missing values for any variable used. |
| Syntax | | REGRESSION  /DESCRIPTIVES MEAN STDDEV CORR SIG N  /MISSING LISTWISE  /STATISTICS COEFF OUTS R ANOVA CHANGE  /CRITERIA=PIN(.05) POUT(.10)  /NOORIGIN  /DEPENDENT P3O  /METHOD=ENTER P3P  /RESIDUALS DURBIN. |
| Resources | Processor Time | 00 00:00:00.016 |
|  | Elapsed Time | 00 00:00:00.031 |
|  | Memory Required | 1796 bytes |
|  | Additional Memory Required for Residual Plots | 0 bytes |

[DataSet0]

| **Descriptive Statistics** | | | |
| --- | --- | --- | --- |
|  | Mean | Std. Deviation | N |
| P3O | 24.0039 | 4.81885 | 144 |
| P3P | 23.9667 | 4.78299 | 144 |

| **Correlations** | | | |
| --- | --- | --- | --- |
|  | | P3O | P3P |
| Pearson Correlation | P3O | 1.000 | .991 |
|  | P3P | .991 | 1.000 |
| Sig. (1-tailed) | P3O | . | .000 |
|  | P3P | .000 | . |
| N | P3O | 144 | 144 |
|  | P3P | 144 | 144 |

| **Variables Entered/Removed^b^** | | | |
| --- | --- | --- | --- |
| Model | Variables Entered | Variables Removed | Method |
| 1 | P3P^a^ | . | Enter |
| a. All requested variables entered.  b. Dependent Variable: P3O | | | |

| **Model Summary^b^** | | | | |
| --- | --- | --- | --- | --- |
| Model | R | R Square | Adjusted R Square | Std. Error of the Estimate |
| 1 | .991^a^ | .981 | .981 | .65862 |

| **Model Summary^b^** | | | | | | |
| --- | --- | --- | --- | --- | --- | --- |
| Model | Change Statistics | | | | | Durbin-Watson |
|  | R Square Change | F Change | df1 | df2 | Sig. F Change |  |
| 1 | .981 | 7513.245 | 1 | 142 | .000 | 2.555 |

|  |
| --- |
| a. Predictors: (Constant), P3P  b. Dependent Variable: P3O |

| **ANOVA^b^** | | | | | | |
| --- | --- | --- | --- | --- | --- | --- |
| Model | | Sum of Squares | df | Mean Square | F | Sig. |
| 1 | Regression | 3259.051 | 1 | 3259.051 | 7513.245 | .000^a^ |
|  | Residual | 61.596 | 142 | .434 |  |  |
|  | Total | 3320.647 | 143 |  |  |  |
| a. Predictors: (Constant), P3P  b. Dependent Variable: P3O | | | | | | |

| **Coefficients^a^** | | | | | | |
| --- | --- | --- | --- | --- | --- | --- |
| Model | | Unstandardized Coefficients | | Standardized Coefficients | t | Sig. |
|  |  | B | Std. Error | Beta |  |  |
| 1 | (Constant) | .083 | .281 |  | .293 | .770 |
|  | P3P | .998 | .012 | .991 | 86.679 | .000 |
| a. Dependent Variable: P3O | | | | | | |

| **Residuals Statistics^a^** | | | | | |
| --- | --- | --- | --- | --- | --- |
|  | Minimum | Maximum | Mean | Std. Deviation | N |
| Predicted Value | 14.6048 | 32.8749 | 24.0039 | 4.77395 | 144 |
| Residual | -3.34832 | 3.96359 | .00000 | .65631 | 144 |
| Std. Predicted Value | -1.969 | 1.858 | .000 | 1.000 | 144 |
| Std. Residual | -5.084 | 6.018 | .000 | .996 | 144 |
| a. Dependent Variable: P3O | | | | | |

REGRESSION

/DESCRIPTIVES MEAN STDDEV CORR SIG N

/MISSING LISTWISE

/STATISTICS COEFF OUTS R ANOVA CHANGE

/CRITERIA=PIN(.05) POUT(.10)

/NOORIGIN

/DEPENDENT P4O

/METHOD=ENTER P4P

/RESIDUALS DURBIN.

**Regression**

| **Notes** | | |
| --- | --- | --- |
| Output Created | | 13-Jan-2021 05:38:49 |
| Comments | |  |
| Input | Active Dataset | DataSet0 |
|  | Filter | <none> |
|  | Weight | <none> |
|  | Split File | <none> |
|  | N of Rows in Working Data File | 144 |
| Missing Value Handling | Definition of Missing | User-defined missing values are treated as missing. |
|  | Cases Used | Statistics are based on cases with no missing values for any variable used. |
| Syntax | | REGRESSION  /DESCRIPTIVES MEAN STDDEV CORR SIG N  /MISSING LISTWISE  /STATISTICS COEFF OUTS R ANOVA CHANGE  /CRITERIA=PIN(.05) POUT(.10)  /NOORIGIN  /DEPENDENT P4O  /METHOD=ENTER P4P  /RESIDUALS DURBIN. |
| Resources | Processor Time | 00 00:00:00.031 |
|  | Elapsed Time | 00 00:00:00.110 |
|  | Memory Required | 1796 bytes |
|  | Additional Memory Required for Residual Plots | 0 bytes |

[DataSet0]

| **Descriptive Statistics** | | | |
| --- | --- | --- | --- |
|  | Mean | Std. Deviation | N |
| P4O | 21.6840 | 7.89048 | 144 |
| P4P | 21.7057 | 7.81685 | 144 |

| **Correlations** | | | |
| --- | --- | --- | --- |
|  | | P4O | P4P |
| Pearson Correlation | P4O | 1.000 | .997 |
|  | P4P | .997 | 1.000 |
| Sig. (1-tailed) | P4O | . | .000 |
|  | P4P | .000 | . |
| N | P4O | 144 | 144 |
|  | P4P | 144 | 144 |

| **Variables Entered/Removed^b^** | | | |
| --- | --- | --- | --- |
| Model | Variables Entered | Variables Removed | Method |
| 1 | P4P^a^ | . | Enter |
| a. All requested variables entered.  b. Dependent Variable: P4O | | | |

| **Model Summary^b^** | | | | |
| --- | --- | --- | --- | --- |
| Model | R | R Square | Adjusted R Square | Std. Error of the Estimate |
| 1 | .997^a^ | .995 | .995 | .58016 |

| **Model Summary^b^** | | | | | | |
| --- | --- | --- | --- | --- | --- | --- |
| Model | Change Statistics | | | | | Durbin-Watson |
|  | R Square Change | F Change | df1 | df2 | Sig. F Change |  |
| 1 | .995 | 26309.729 | 1 | 142 | .000 | 1.995 |

|  |
| --- |
| a. Predictors: (Constant), P4P  b. Dependent Variable: P4O |

| **ANOVA^b^** | | | | | | |
| --- | --- | --- | --- | --- | --- | --- |
| Model | | Sum of Squares | df | Mean Square | F | Sig. |
| 1 | Regression | 8855.332 | 1 | 8855.332 | 26309.729 | .000^a^ |
|  | Residual | 47.794 | 142 | .337 |  |  |
|  | Total | 8903.127 | 143 |  |  |  |
| a. Predictors: (Constant), P4P  b. Dependent Variable: P4O | | | | | | |

| **Coefficients^a^** | | | | | | |
| --- | --- | --- | --- | --- | --- | --- |
| Model | | Unstandardized Coefficients | | Standardized Coefficients | t | Sig. |
|  |  | B | Std. Error | Beta |  |  |
| 1 | (Constant) | -.167 | .143 |  | -1.168 | .245 |
|  | P4P | 1.007 | .006 | .997 | 162.203 | .000 |
| a. Dependent Variable: P4O | | | | | | |

| **Residuals Statistics^a^** | | | | | |
| --- | --- | --- | --- | --- | --- |
|  | Minimum | Maximum | Mean | Std. Deviation | N |
| Predicted Value | 9.6686 | 55.6937 | 21.6840 | 7.86927 | 144 |
| Residual | -3.28557 | 2.65526 | .00000 | .57812 | 144 |
| Std. Predicted Value | -1.527 | 4.322 | .000 | 1.000 | 144 |
| Std. Residual | -5.663 | 4.577 | .000 | .996 | 144 |
| a. Dependent Variable: P4O | | | | | |

REGRESSION

/DESCRIPTIVES MEAN STDDEV CORR SIG N

/MISSING LISTWISE

/STATISTICS COEFF OUTS R ANOVA CHANGE

/CRITERIA=PIN(.05) POUT(.10)

/NOORIGIN

/DEPENDENT K1O

/METHOD=ENTER K1P

/RESIDUALS DURBIN.

**Regression**

| **Notes** | | |
| --- | --- | --- |
| Output Created | | 13-Jan-2021 05:39:15 |
| Comments | |  |
| Input | Active Dataset | DataSet0 |
|  | Filter | <none> |
|  | Weight | <none> |
|  | Split File | <none> |
|  | N of Rows in Working Data File | 144 |
| Missing Value Handling | Definition of Missing | User-defined missing values are treated as missing. |
|  | Cases Used | Statistics are based on cases with no missing values for any variable used. |
| Syntax | | REGRESSION  /DESCRIPTIVES MEAN STDDEV CORR SIG N  /MISSING LISTWISE  /STATISTICS COEFF OUTS R ANOVA CHANGE  /CRITERIA=PIN(.05) POUT(.10)  /NOORIGIN  /DEPENDENT K1O  /METHOD=ENTER K1P  /RESIDUALS DURBIN. |
| Resources | Processor Time | 00 00:00:00.016 |
|  | Elapsed Time | 00 00:00:00.093 |
|  | Memory Required | 1796 bytes |
|  | Additional Memory Required for Residual Plots | 0 bytes |

[DataSet0]

| **Descriptive Statistics** | | | |
| --- | --- | --- | --- |
|  | Mean | Std. Deviation | N |
| K1O | 14.3452 | 2.68643 | 144 |
| K1P | 14.2749 | 2.43405 | 144 |

| **Correlations** | | | |
| --- | --- | --- | --- |
|  | | K1O | K1P |
| Pearson Correlation | K1O | 1.000 | .970 |
|  | K1P | .970 | 1.000 |
| Sig. (1-tailed) | K1O | . | .000 |
|  | K1P | .000 | . |
| N | K1O | 144 | 144 |
|  | K1P | 144 | 144 |

| **Variables Entered/Removed^b^** | | | |
| --- | --- | --- | --- |
| Model | Variables Entered | Variables Removed | Method |
| 1 | K1P^a^ | . | Enter |
| a. All requested variables entered.  b. Dependent Variable: K1O | | | |

| **Model Summary^b^** | | | | |
| --- | --- | --- | --- | --- |
| Model | R | R Square | Adjusted R Square | Std. Error of the Estimate |
| 1 | .970^a^ | .940 | .940 | .65979 |

| **Model Summary^b^** | | | | | | |
| --- | --- | --- | --- | --- | --- | --- |
| Model | Change Statistics | | | | | Durbin-Watson |
|  | R Square Change | F Change | df1 | df2 | Sig. F Change |  |
| 1 | .940 | 2228.673 | 1 | 142 | .000 | 1.892 |

|  |
| --- |
| a. Predictors: (Constant), K1P  b. Dependent Variable: K1O |

| **ANOVA^b^** | | | | | | |
| --- | --- | --- | --- | --- | --- | --- |
| Model | | Sum of Squares | df | Mean Square | F | Sig. |
| 1 | Regression | 970.200 | 1 | 970.200 | 2228.673 | .000^a^ |
|  | Residual | 61.816 | 142 | .435 |  |  |
|  | Total | 1032.017 | 143 |  |  |  |
| a. Predictors: (Constant), K1P  b. Dependent Variable: K1O | | | | | | |

| **Coefficients^a^** | | | | | | |
| --- | --- | --- | --- | --- | --- | --- |
| Model | | Unstandardized Coefficients | | Standardized Coefficients | t | Sig. |
|  |  | B | Std. Error | Beta |  |  |
| 1 | (Constant) | -.931 | .328 |  | -2.836 | .005 |
|  | K1P | 1.070 | .023 | .970 | 47.209 | .000 |
| a. Dependent Variable: K1O | | | | | | |

| **Residuals Statistics^a^** | | | | | |
| --- | --- | --- | --- | --- | --- |
|  | Minimum | Maximum | Mean | Std. Deviation | N |
| Predicted Value | 9.5779 | 23.3931 | 14.3452 | 2.60473 | 144 |
| Residual | -1.59079 | 4.28716 | .00000 | .65748 | 144 |
| Std. Predicted Value | -1.830 | 3.474 | .000 | 1.000 | 144 |
| Std. Residual | -2.411 | 6.498 | .000 | .996 | 144 |
| a. Dependent Variable: K1O | | | | | |

REGRESSION

/DESCRIPTIVES MEAN STDDEV CORR SIG N

/MISSING LISTWISE

/STATISTICS COEFF OUTS R ANOVA CHANGE

/CRITERIA=PIN(.05) POUT(.10)

/NOORIGIN

/DEPENDENT K2O

/METHOD=ENTER K2P

/RESIDUALS DURBIN.

**Regression**

| **Notes** | | |
| --- | --- | --- |
| Output Created | | 13-Jan-2021 05:44:47 |
| Comments | |  |
| Input | Active Dataset | DataSet0 |
|  | Filter | <none> |
|  | Weight | <none> |
|  | Split File | <none> |
|  | N of Rows in Working Data File | 144 |
| Missing Value Handling | Definition of Missing | User-defined missing values are treated as missing. |
|  | Cases Used | Statistics are based on cases with no missing values for any variable used. |
| Syntax | | REGRESSION  /DESCRIPTIVES MEAN STDDEV CORR SIG N  /MISSING LISTWISE  /STATISTICS COEFF OUTS R ANOVA CHANGE  /CRITERIA=PIN(.05) POUT(.10)  /NOORIGIN  /DEPENDENT K2O  /METHOD=ENTER K2P  /RESIDUALS DURBIN. |
| Resources | Processor Time | 00 00:00:00.016 |
|  | Elapsed Time | 00 00:00:00.031 |
|  | Memory Required | 1796 bytes |
|  | Additional Memory Required for Residual Plots | 0 bytes |

[DataSet0]

| **Descriptive Statistics** | | | |
| --- | --- | --- | --- |
|  | Mean | Std. Deviation | N |
| K2O | 16.8314 | 2.54017 | 144 |
| K2P | 16.7845 | 2.40673 | 144 |

| **Correlations** | | | |
| --- | --- | --- | --- |
|  | | K2O | K2P |
| Pearson Correlation | K2O | 1.000 | .984 |
|  | K2P | .984 | 1.000 |
| Sig. (1-tailed) | K2O | . | .000 |
|  | K2P | .000 | . |
| N | K2O | 144 | 144 |
|  | K2P | 144 | 144 |

| **Variables Entered/Removed^b^** | | | |
| --- | --- | --- | --- |
| Model | Variables Entered | Variables Removed | Method |
| 1 | K2P^a^ | . | Enter |
| a. All requested variables entered.  b. Dependent Variable: K2O | | | |

| **Model Summary^b^** | | | | |
| --- | --- | --- | --- | --- |
| Model | R | R Square | Adjusted R Square | Std. Error of the Estimate |
| 1 | .984^a^ | .968 | .968 | .45677 |

| **Model Summary^b^** | | | | | | |
| --- | --- | --- | --- | --- | --- | --- |
| Model | Change Statistics | | | | | Durbin-Watson |
|  | R Square Change | F Change | df1 | df2 | Sig. F Change |  |
| 1 | .968 | 4280.426 | 1 | 142 | .000 | 1.914 |

|  |
| --- |
| a. Predictors: (Constant), K2P  b. Dependent Variable: K2O |

| **ANOVA^b^** | | | | | | |
| --- | --- | --- | --- | --- | --- | --- |
| Model | | Sum of Squares | df | Mean Square | F | Sig. |
| 1 | Regression | 893.072 | 1 | 893.072 | 4280.426 | .000^a^ |
|  | Residual | 29.627 | 142 | .209 |  |  |
|  | Total | 922.699 | 143 |  |  |  |
| a. Predictors: (Constant), K2P  b. Dependent Variable: K2O | | | | | | |

| **Coefficients^a^** | | | | | | |
| --- | --- | --- | --- | --- | --- | --- |
| Model | | Unstandardized Coefficients | | Standardized Coefficients | t | Sig. |
|  |  | B | Std. Error | Beta |  |  |
| 1 | (Constant) | -.597 | .269 |  | -2.219 | .028 |
|  | K2P | 1.038 | .016 | .984 | 65.425 | .000 |
| a. Dependent Variable: K2O | | | | | | |

| **Residuals Statistics^a^** | | | | | |
| --- | --- | --- | --- | --- | --- |
|  | Minimum | Maximum | Mean | Std. Deviation | N |
| Predicted Value | 12.3098 | 22.8907 | 16.8314 | 2.49905 | 144 |
| Residual | -1.74066 | 2.68190 | .00000 | .45517 | 144 |
| Std. Predicted Value | -1.809 | 2.425 | .000 | 1.000 | 144 |
| Std. Residual | -3.811 | 5.871 | .000 | .996 | 144 |
| a. Dependent Variable: K2O | | | | | |

REGRESSION

/DESCRIPTIVES MEAN STDDEV CORR SIG N

/MISSING LISTWISE

/STATISTICS COEFF OUTS R ANOVA CHANGE

/CRITERIA=PIN(.05) POUT(.10)

/NOORIGIN

/DEPENDENT K3O

/METHOD=ENTER K3P

/RESIDUALS DURBIN.

**Regression**

| **Notes** | | |
| --- | --- | --- |
| Output Created | | 13-Jan-2021 05:45:13 |
| Comments | |  |
| Input | Active Dataset | DataSet0 |
|  | Filter | <none> |
|  | Weight | <none> |
|  | Split File | <none> |
|  | N of Rows in Working Data File | 144 |
| Missing Value Handling | Definition of Missing | User-defined missing values are treated as missing. |
|  | Cases Used | Statistics are based on cases with no missing values for any variable used. |
| Syntax | | REGRESSION  /DESCRIPTIVES MEAN STDDEV CORR SIG N  /MISSING LISTWISE  /STATISTICS COEFF OUTS R ANOVA CHANGE  /CRITERIA=PIN(.05) POUT(.10)  /NOORIGIN  /DEPENDENT K3O  /METHOD=ENTER K3P  /RESIDUALS DURBIN. |
| Resources | Processor Time | 00 00:00:00.016 |
|  | Elapsed Time | 00 00:00:00.031 |
|  | Memory Required | 1796 bytes |
|  | Additional Memory Required for Residual Plots | 0 bytes |

[DataSet0]

| **Descriptive Statistics** | | | |
| --- | --- | --- | --- |
|  | Mean | Std. Deviation | N |
| K3O | 9.0990 | 1.83319 | 144 |
| K3P | 9.0962 | 1.79242 | 144 |

| **Correlations** | | | |
| --- | --- | --- | --- |
|  | | K3O | K3P |
| Pearson Correlation | K3O | 1.000 | .992 |
|  | K3P | .992 | 1.000 |
| Sig. (1-tailed) | K3O | . | .000 |
|  | K3P | .000 | . |
| N | K3O | 144 | 144 |
|  | K3P | 144 | 144 |

| **Variables Entered/Removed^b^** | | | |
| --- | --- | --- | --- |
| Model | Variables Entered | Variables Removed | Method |
| 1 | K3P^a^ | . | Enter |
| a. All requested variables entered.  b. Dependent Variable: K3O | | | |

| **Model Summary^b^** | | | | |
| --- | --- | --- | --- | --- |
| Model | R | R Square | Adjusted R Square | Std. Error of the Estimate |
| 1 | .992^a^ | .984 | .984 | .22953 |

| **Model Summary^b^** | | | | | | |
| --- | --- | --- | --- | --- | --- | --- |
| Model | Change Statistics | | | | | Durbin-Watson |
|  | R Square Change | F Change | df1 | df2 | Sig. F Change |  |
| 1 | .984 | 8979.566 | 1 | 142 | .000 | 2.095 |

|  |
| --- |
| a. Predictors: (Constant), K3P  b. Dependent Variable: K3O |

| **ANOVA^b^** | | | | | | |
| --- | --- | --- | --- | --- | --- | --- |
| Model | | Sum of Squares | df | Mean Square | F | Sig. |
| 1 | Regression | 473.085 | 1 | 473.085 | 8979.566 | .000^a^ |
|  | Residual | 7.481 | 142 | .053 |  |  |
|  | Total | 480.566 | 143 |  |  |  |
| a. Predictors: (Constant), K3P  b. Dependent Variable: K3O | | | | | | |

| **Coefficients^a^** | | | | | | |
| --- | --- | --- | --- | --- | --- | --- |
| Model | | Unstandardized Coefficients | | Standardized Coefficients | t | Sig. |
|  |  | B | Std. Error | Beta |  |  |
| 1 | (Constant) | -.131 | .099 |  | -1.324 | .188 |
|  | K3P | 1.015 | .011 | .992 | 94.761 | .000 |
| a. Dependent Variable: K3O | | | | | | |

| **Residuals Statistics^a^** | | | | | |
| --- | --- | --- | --- | --- | --- |
|  | Minimum | Maximum | Mean | Std. Deviation | N |
| Predicted Value | 5.7541 | 13.8199 | 9.0990 | 1.81887 | 144 |
| Residual | -1.87141 | .81183 | .00000 | .22873 | 144 |
| Std. Predicted Value | -1.839 | 2.596 | .000 | 1.000 | 144 |
| Std. Residual | -8.153 | 3.537 | .000 | .996 | 144 |
| a. Dependent Variable: K3O | | | | | |

REGRESSION

/DESCRIPTIVES MEAN STDDEV CORR SIG N

/MISSING LISTWISE

/STATISTICS COEFF OUTS R ANOVA CHANGE

/CRITERIA=PIN(.05) POUT(.10)

/NOORIGIN

/DEPENDENT K4O

/METHOD=ENTER K4P

/RESIDUALS DURBIN.

**Regression**

| **Notes** | | |
| --- | --- | --- |
| Output Created | | 13-Jan-2021 05:45:51 |
| Comments | |  |
| Input | Active Dataset | DataSet0 |
|  | Filter | <none> |
|  | Weight | <none> |
|  | Split File | <none> |
|  | N of Rows in Working Data File | 144 |
| Missing Value Handling | Definition of Missing | User-defined missing values are treated as missing. |
|  | Cases Used | Statistics are based on cases with no missing values for any variable used. |
| Syntax | | REGRESSION  /DESCRIPTIVES MEAN STDDEV CORR SIG N  /MISSING LISTWISE  /STATISTICS COEFF OUTS R ANOVA CHANGE  /CRITERIA=PIN(.05) POUT(.10)  /NOORIGIN  /DEPENDENT K4O  /METHOD=ENTER K4P  /RESIDUALS DURBIN. |
| Resources | Processor Time | 00 00:00:00.031 |
|  | Elapsed Time | 00 00:00:00.173 |
|  | Memory Required | 1796 bytes |
|  | Additional Memory Required for Residual Plots | 0 bytes |

[DataSet0]

| **Descriptive Statistics** | | | |
| --- | --- | --- | --- |
|  | Mean | Std. Deviation | N |
| K4O | 11.9165 | 3.18262 | 144 |
| K4P | 11.8062 | 3.10607 | 144 |

| **Correlations** | | | |
| --- | --- | --- | --- |
|  | | K4O | K4P |
| Pearson Correlation | K4O | 1.000 | .976 |
|  | K4P | .976 | 1.000 |
| Sig. (1-tailed) | K4O | . | .000 |
|  | K4P | .000 | . |
| N | K4O | 144 | 144 |
|  | K4P | 144 | 144 |

| **Variables Entered/Removed^b^** | | | |
| --- | --- | --- | --- |
| Model | Variables Entered | Variables Removed | Method |
| 1 | K4P^a^ | . | Enter |
| a. All requested variables entered.  b. Dependent Variable: K4O | | | |

| **Model Summary^b^** | | | | |
| --- | --- | --- | --- | --- |
| Model | R | R Square | Adjusted R Square | Std. Error of the Estimate |
| 1 | .976^a^ | .952 | .952 | .69907 |

| **Model Summary^b^** | | | | | | |
| --- | --- | --- | --- | --- | --- | --- |
| Model | Change Statistics | | | | | Durbin-Watson |
|  | R Square Change | F Change | df1 | df2 | Sig. F Change |  |
| 1 | .952 | 2821.907 | 1 | 142 | .000 | 2.149 |

|  |
| --- |
| a. Predictors: (Constant), K4P  b. Dependent Variable: K4O |

| **ANOVA^b^** | | | | | | |
| --- | --- | --- | --- | --- | --- | --- |
| Model | | Sum of Squares | df | Mean Square | F | Sig. |
| 1 | Regression | 1379.059 | 1 | 1379.059 | 2821.907 | .000^a^ |
|  | Residual | 69.395 | 142 | .489 |  |  |
|  | Total | 1448.454 | 143 |  |  |  |
| a. Predictors: (Constant), K4P  b. Dependent Variable: K4O | | | | | | |

| **Coefficients^a^** | | | | | | |
| --- | --- | --- | --- | --- | --- | --- |
| Model | | Unstandardized Coefficients | | Standardized Coefficients | t | Sig. |
|  |  | B | Std. Error | Beta |  |  |
| 1 | (Constant) | .113 | .230 |  | .491 | .624 |
|  | K4P | 1.000 | .019 | .976 | 53.122 | .000 |
| a. Dependent Variable: K4O | | | | | | |

| **Residuals Statistics^a^** | | | | | |
| --- | --- | --- | --- | --- | --- |
|  | Minimum | Maximum | Mean | Std. Deviation | N |
| Predicted Value | 5.5816 | 18.9989 | 11.9165 | 3.10544 | 144 |
| Residual | -1.65920 | 4.45774 | .00000 | .69662 | 144 |
| Std. Predicted Value | -2.040 | 2.281 | .000 | 1.000 | 144 |
| Std. Residual | -2.373 | 6.377 | .000 | .996 | 144 |
| a. Dependent Variable: K4O | | | | | |
